# dHis-Cu(ii) based EPR distances at W-band: navigating the challenges of excitation

**DOI:** 10.1039/d6cp02541b

**Published:** 2026-09-14

**Authors:** Nicholas A. Moriglioni, Yannik Limbach, Hassane El Mkami, Robert I. Hunter, Katrin Ackermann, Graham M. Smith, Bela E. Bode, Sunil Saxena

**Affiliations:** a Department of Chemistry, University of Pittsburgh PA 15260 USA sksaxena@pitt.edu; b EaStCHEM School of Chemistry, Biomedical Sciences Research Complex and Centre of Magnetic Resonance, University of St. Andrews North Haugh St Andrews KY16 9ST Scotland UK beb2@st-andrews.ac.uk; c Clausius-Institute for Physical and Theoretical Chemistry, University of Bonn Wegelerstr. 12 53115 Bonn Germany; d SUPA, School of Physics & Astronomy, University of St Andrews North Haugh St Andrews Fife KY16 9SS UK

## Abstract

The measurement of nanometer range distance constraints by pulsed dipolar EPR spectroscopy has become an invaluable tool for integrative biophysics. Combining rigid spin labels, such as Cu(ii)-NTA, with high frequency EPR is attractive to measure structural information with high precision. Such measurements are, however, challenged by limited excitation bandwidth which can lead to complications in both data acquisition and analysis. Herein, we examine the distance measurements on proteins labeled with Cu(ii) at W-band (ca. 95 GHz) frequencies. The large anisotropy of the *g*-tensor leads to a spectral width of about ten GHz. Thus, the acquisition of an orientation independent dipolar trace becomes non-trivial with pulses of three orders of magnitude smaller bandwidth. We show using a recently developed computational approach that acquisition at only four field positions is sufficient to yield orientation averaged distance distributions. This acquisition relies on excitation of only ∼0.43% of spins in total. The computational approach is validated on data from two samples of doubly labeled GB1 protein. We anticipate that this conceptual methodology will be of use for a wide variety of high field pulsed EPR measurements.

## Abstract 2

A computationally designed acquisition scheme captures distance distribution despite the excitation of less than 1% of the Cu(ii)-NTA spins at W-Band EPR.

## Graphical abstract

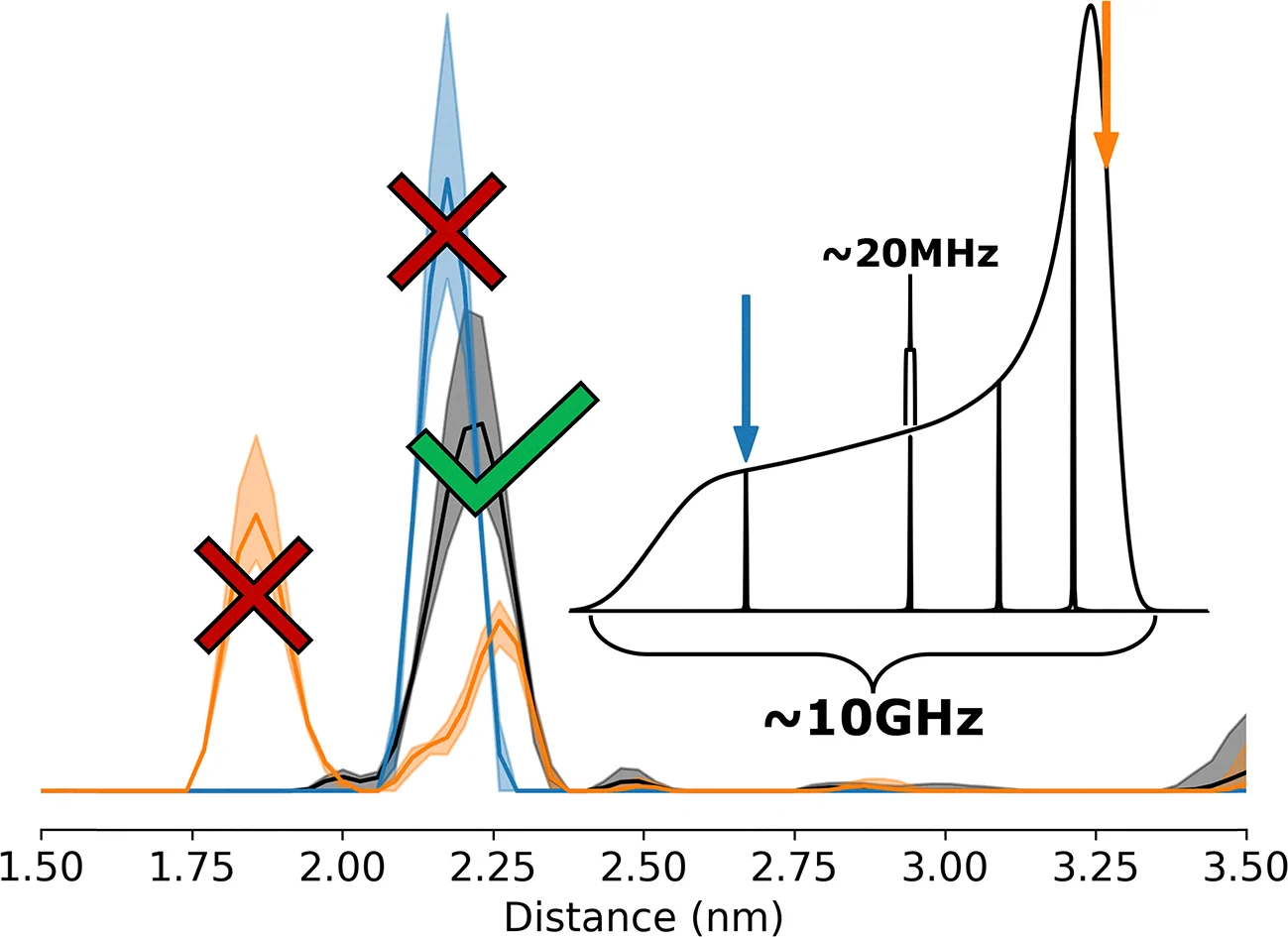

## Introduction

Pulsed dipolar EPR spectroscopy (PDS) has become an invaluable tool for structural biology. This spectroscopy can measure the distance distributions between a pair of spin labels in the range of 15 to 160 Å.^1,2^ The PDS methodology is especially powerful because the full conformational ensemble of a biomolecule is measured in frozen solution, without the need for crystallization. Additionally, PDS is sensitive only to paramagnetic species, which enables the method to focus on the needed structural constraint without complication by signals from the surrounding diamagnetic molecules. Thus, measurements can be made on biomolecules without losing contrast. On the other hand, the method typically requires site-directed spin labeling.^3,4^ PDS has been exploited in cellular environments^5–16^ as well as for membrane proteins.^17–22^ The exclusive sensitivity for paramagnetic species also makes PDS a viable method for the study of large biomolecules.^2,23^

The most used spin label for proteins (MTSSL, giving rise to the modified protein sidechain R1) contains a nitroxide radical as an EPR reporter. The label is attached to proteins *via* a linker that forms a covalent bond with cysteine residues.^3^ Similar chemistry is used for other nitroxide,^24,25^ carbon,^14,20,26–28^ gadolinium,^29^ and some copper-based spin labels.^30^ However, the spin labeling to cysteine is typically achieved by the use of a linker containing five or more rotatable bonds. Hence, distance distributions are broadened beyond the ensemble of backbone conformations due to the rotamer distribution of the spin labels. Consequently, a limitation of distance measurements *via* PDS is the translation of spin–spin distance distributions into an ensemble of backbone conformations.

To obtain distance distributions that more accurately report on the protein backbone, rigid spin labels have been developed. These include labels that use a dual cystine linker,^31^ noncanonical paramagnetic amino acids incorporated during peptide synthesis,^32,33^ as well as transition metal labels that rely on double histidine (dHis) residue coordination.^34,35^ One such label is Cu(ii)-nitrilotriacetic acid (Cu(ii)-NTA).^36^ Such rigid labels produce narrower distance distributions and report on biomolecular distances more precisely than flexible spin labels.^34,37–39^ With more precise distance distribution data, fewer experiments are needed to locate ligand binding sites,^40^ and conformational states can be better resolved.^41–43^ These advantages, however, can come at the expense of sensitivity. Typically, Cu(ii) based distance measurements are performed using 1–5 times higher protein concentrations compared to nitroxide labels,^44^ although single frequency experiments such as RIDME (relaxation-induced dipolar modulation enhancement) provide improved sensitivity.^45^ It is also worth noting that despite the coordinative nature of labelling, Cu(ii)-NTA is a reliable spin label even in the presence of competitor ions, across a range of pH,^46^ and in an array of buffer solutions.^47^ Importantly, the label is selective to dHis sites even in the presence of a large excess of single His sites.^48^ Thus the creation of a His-null protein background is not necessary.

PDS experiments performed at higher frequency benefit from increased concentration sensitivity.^49–51^ Specifically, DEER (double electron-electron resonance also known as PELDOR) measurements on nitroxides at Q-band (∼34.5 GHz) show thirteen times higher SNR (singal-to-noise ratio) compared to those done at X-band (∼9.5 GHz).^49^ Further, W-band (∼94.5 GHz) has been shown to improve sensitivity by a factor of thirty.^52^

Accessing the higher sensitivity is, however, non-trivial for Cu(ii). Higher frequency increases the spectral width of the EPR spectrum. This broadening is especially problematic for Cu(ii)-NTA, due to the much larger *g*-anisotropy of Cu(ii) compared to nitroxides. For Cu(ii)-NTA, the spectral breadth goes from approximately 1.8 GHz at X-band to about 4.5 GHz at Q-band, and to ∼10 GHz at W-band. This broadening reduces the number of spins that are excited by a given pulse bandwidth. Further, the spectral breadth results in selective sampling of spins based on their orientation with respect to the applied magnetic field, a phenomenon known as orientational selectivity. When orientations of the spin and the interspin vector are strongly correlated, orientational selectivity results in non-statistical sampling of interspin vector orientations. Notably, this correlation is weak for the flexible spin labels. Thus, orientational selectivity is often not a concern for R1 labeled proteins, except in cases where the label is buried or otherwise constrained.^53^

However, for dHis bound Cu(ii)-NTA, the correlation between spin orientation and the orientation of the interspin vector is strong. Therefore, the PDS signal can become dependent on both the interspin distance as well as on the orientation of the interspin vectors of the spin pairs that are sampled.^48^ This phenomenon poses challenges to both data acquisition as well as analysis. Such orientational selectivity is also an important concept for other high-frequency pulsed EPR experiments such as electron spin echo envelope modulation (ESEEM),^54,55^ hyperfine sublevel correlation spectroscopy (HYSCORE),^56,57^ and electron nuclear double resonance (ENDOR).^58–62^

In general, two philosophies have emerged to mitigate the effects of orientational selectivity in pulsed experiments. The first approach relies on collection of data at many field positions and summing the data prior to analysis.^63–68^ The entire set of data can also be simulated to decipher information of distance as well as the relative orientations of the spins.^53,64,69,70^ This method comes at the expense of sensitivity, especially for Cu(ii), since the signal can be small at many field positions. In the second approach the data is acquired only at canonical field positions such as at field positions corresponding to resonances at *g*_*xx*_, *g*_*yy*_, and *g*_*zz*_.^71–78^ This approach, besides being empirical, ignores the spread of resonances due to hyperfine interactions, which can lead to imperfect excitation.

We have recently developed a conceptual framework that provides detailed orientational insights into pulsed excitation. Based on this computational framework we have shown that summing the data acquired at two or three field positions (depending on spectrometer configurations) at Q-band is sufficient to measure the distance distribution from dHis-Cu(ii) labeled proteins independent of mutual orientation of the labelling sites.^79,80^ On the other hand, due to the nature of dHis-Cu(ii) coordination the mitigation of orientation effects at Q-band has modest requirements.

This work achieves the ultimate test of the conceptual framework by establishing the acquisition scheme for RIDME measurements on dHis-Cu(ii) at W-band when limited to relatively narrow bandwidth pulses. The spectral width of the label at W-band is *ca.* 10 GHz, whereas the pulse used herein excite less than 1% of the spectrum. Despite the limited excitation, our computations show that only four to five field positions are required for acquisition of orientationally averaged PDS data. Remarkably, none of these fields are at canonical *g*-tensor positions. The idea is experimentally validated on two different protein samples.

The paper is structured as follows. We first describe the computational strategy, including improvements in methodology from prior work. The key considerations for computational modeling are discussed next, and finally we present the data on two protein samples.

## Experimental methodology

### Protein purification and EPR sample preparation

The E15H/T17H/K28H/Q32H and I6H/N8H/K28H/Q32H mutants of the immunoglobulin-binding B1 domain of group G streptococcal protein G (GB1) were purified as described previously.^81,82^ Purity of the protein was confirmed *via* electrospray ionization (ESI) mass spectrometry. Labelling with Cu(ii)-NTA was performed as described earlier.^36,81,83^

For HiPER experiments, samples with a final volume of 100 µL were prepared with 500 µM of the respective GB1 constructs, a 1:2 excess of Cu(ii)-NTA in deuterated phosphate buffer (42.4 mM Na_2_HPO_4_, 7.6 mM KH_2_PO_4_, 150 mM NaCl, pH 7.4) and 50% (v/v) deuterated ethylene glycol (Sigma-Aldrich) for cryoprotection. The samples were transferred into fluorinated ethylene propylene (FEP) tubes *via* a Hamilton tubing and flash frozen in liquid nitrogen. FEP tubes are used with samples to avoid sample cracking at low temperatures previously seen with quartz tubes.^84^

### Pulsed EPR measurements

The HiPER W-band spectrometer has been described before^85^ but has since been upgraded to the previously published^84^ state with a two channel 12 GSa s^−1^ M8190A AWG to provide up to 1 GHz instantaneous bandwidth. Samples are typically loaded (and removed) at a spectrometer temperature of 180 K and take about one hour to stabilize at ≈30 K. The temperature was maintained with liquid helium and kept as close to 30 K as possible with the utilized setup.

The Field-Swept Electron Spin Echo detected (FS ESE) EPR spectra were obtained using the pulse sequence (Hahn-Echo): π/2-*τ*-π-*τ*-echo sweeping the magnetic field at 30 K. The π/2 and π pulses were rectangular with a length of 8/16 ns and the interpulse delay *τ* was set to 300 ns. A field range of 3740 G was swept in steps of 10 G. The utilized shot repetition time (SRT) was 200 µs while 500 shots per point were recorded (SPP). A two-step phase cycle was used.

### RIDME measurements

We chose RIDME in this work because DEER on pairs of Cu(ii)-NTA spin labels at W-Band yields a prohibitively low modulation depth, making the technique impractical at low fields. The five pulse RIDME experiments^86^ were set up based on the following pulse sequence: π/2-*τ*_1_-π-(*τ*_1_ + *t*)-π/2-*T*_mix_-π/2-(*τ*_2_ − *t*)-π-*τ*_2_-echo. All RIDME measurements were conducted at 30 K at varying field positions. The π/2 and π pulses were rectangular with a length of 24/48 ns and the first interpulse delay *τ*_1_ was set to 300 ns. Soft pulses were used to avoid pronounced effects from deuterium ESEEM.^87^ The second interpulse delay *τ*_2_ was set to 1200 ns and the acquisition was started 100 ns (*t*) before the expected maximum of the trace while recording with a step size of 8 ns. To adjust the mixing time *T*_mix_ and the shot repetition time (SRT), relaxation time measurements were conducted. For homo spin pairs the optimal *T*_mix_ should be 0.7 × *T*_1_^81^ and has been determined to be 30 µs, fitting mono-exponential decays to the relaxation data. The SRT was set to 100 µs to maximize signal to noise with >80% echo recovery, while 2500 shots per point were recorded. Additionally, 8-step phase cycling was applied, and the signals were typically down-converted to an offset frequency of 200 MHz, Fourier transformed and then digitally filtered in frequency space to remove DC offsets.

The resulting RIDME time traces were normalized to the intensity of the FS ESE spectra and summed up according to the different utilized acquisition schemes. The resulting data and individual traces were analyzed with a modified version of the Tikhonov validation functionality in DeerAnalysis 2022^88^ as previously reported.^89^

## Computational methodology

### Overview of the *in silico* model

**Fig. 1:**
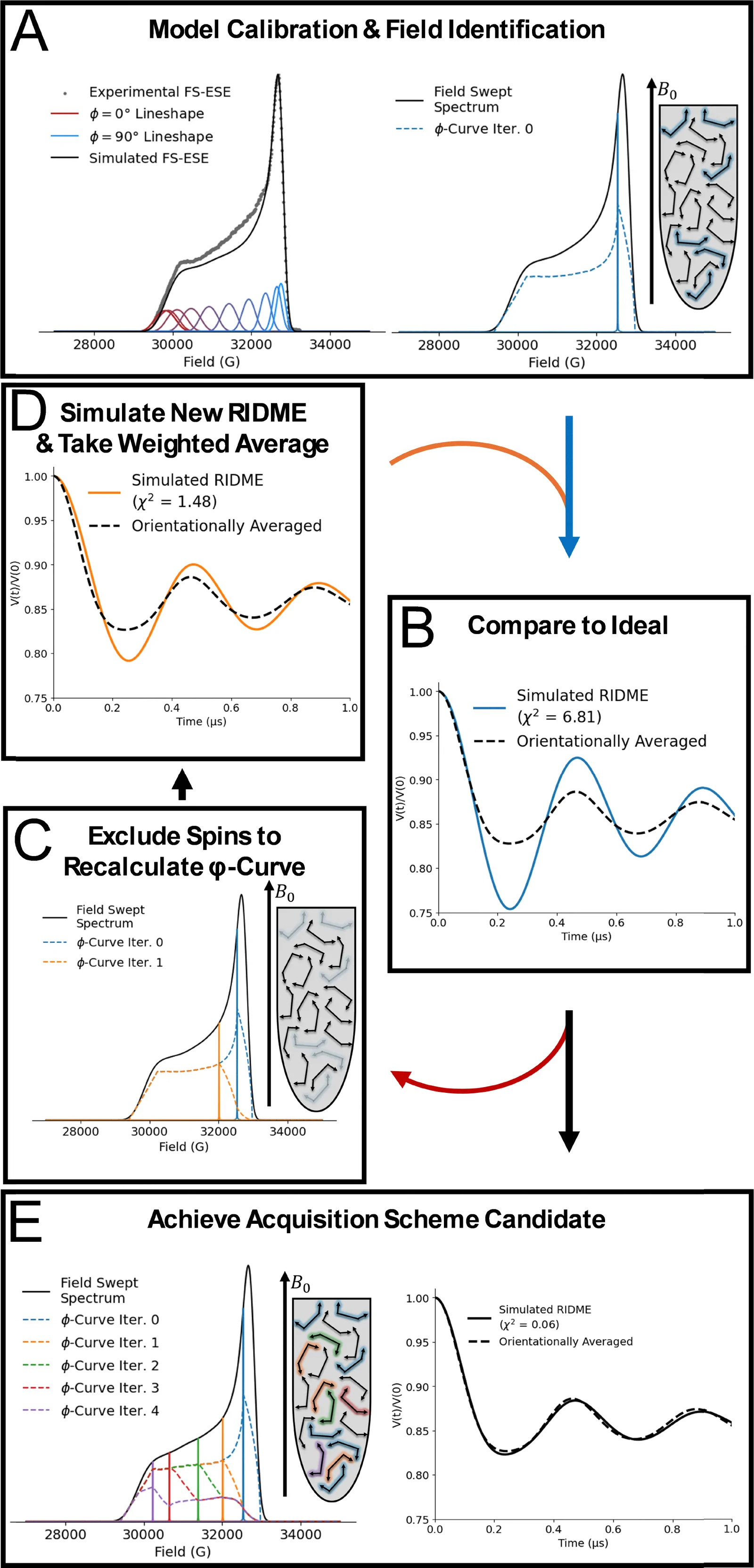
A flowchart that shows the simulation strategy. (A) (Left) A scatter plot of the experimentally collected FS-ESE (grey) overlaid on the simulated FS-ESE (solid, black). Individual spin spectra are portrayed on a red to blue gradient. (Right) The simulated FS-ESE (solid, black) with the 0^th^ iteration of the *ϕ*-curve (dashed, blue). A 48 ns rectangular pulse (solid, blue) is illustrated centered at the maximum of the *ϕ*-curve. A graphic of the *in silico* sample displays each spin pair model (black) with spin pairs excited by the 48 ns pulse (highlighted in blue). (B) The simulated RIDME signal obtained at the field corresponding to the maximum of the *ϕ*-curve (solid, blue) compared against the orientationally averaged ideal signal (dashed, black). (C) The first iteration *ϕ*-curve (gold). A pulse is centered on the new *ϕ*-curve maximum (solid, orange), which is generated by excluding the previously excited spin pairs. (D) The weighted average of all previous RIDME simulations (solid, orange) compared against the ideal signal (dashed, black). (E) (Left) 5 iterations of *ϕ*-curve produced by cycles of steps B through D (dashed blue, gold, green, red, and violet), with maximum field positions identified. (Center) A diagram of the *in silico* test tube indicates which spin pairs have been selected by each simulated RIDME as indicated by the colored highlight. (Right) The simulated RIDME time trace from the 5-field acquisition scheme (solid, black) compared to the ideal time trace (dashed, black).

We illustrate the generalized procedure used to establish the acquisition scheme in Fig. 1. More details of each step are provided in subsequent paragraphs. First, we create an *in silico* sample consisting of an ensemble of spin pairs (*cf.*Fig. 1A). Each spin pair is oriented randomly with respect to *B*_0_. In the first step, each spin is interrogated to calculate its resonance frequencies, and these are summed to create a FS-ESE spectrum. Fig. 1A shows an example of the calculated spectrum overlaid on experimental data. Next, the number of excited spin orientations at each field is tabulated. This resultant curve, known as the *ϕ*-curve, is shown as a blue trace on the right panel of Fig. 1A. We then simulate a RIDME experiment at the maximum of the *ϕ*-curve and compare the simulated RIDME signal to the orientationally averaged signal (*cf.*Fig. 1B). If the simulated RIDME signal differs from the ideal, the *ϕ*-curve is recalculated by omitting the previously excited spin pairs to find the next best sampling position (*cf.*Fig. 1C). The field at the maximum of this new *ϕ*-curve is noted and the process of calculation of RIDME and elimination of excited spin pairs is repeated until five fields are identified. Then the weighted average of different combinations of RIDME are calculated and compared to the ideal orientationally averaged signal (*cf.*Fig. 1D). This process is performed for different relative orientations of *g*-tensors and an acquisition scheme is established when orientationally averaged signal is achieved for all orientations (*cf.*Fig. 1E).

### Creation of the Cu(ii)-NTA W-band *in silico* sample

**Fig. 2:**
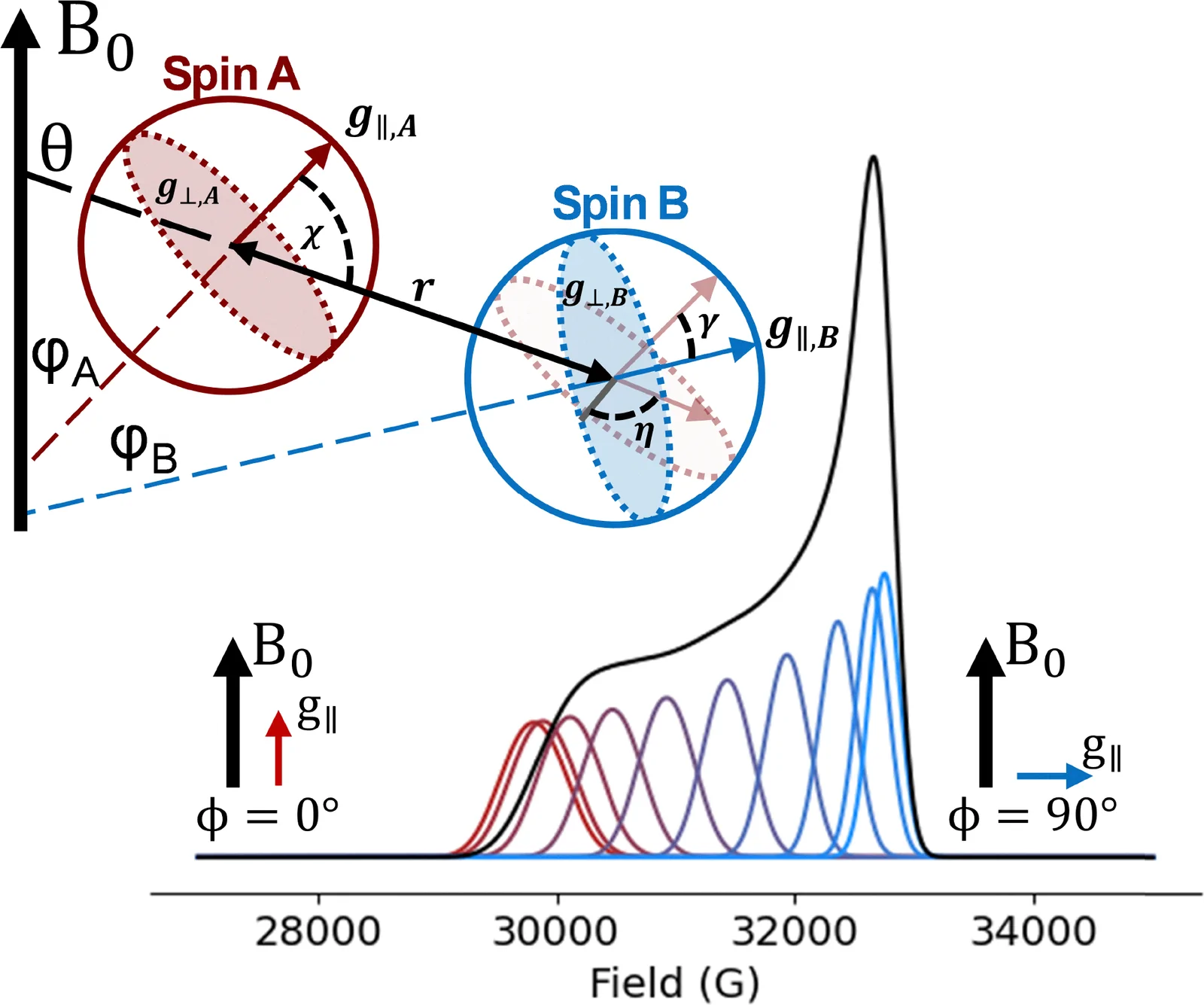
The simulated FS-ESE (black) overlaid on the spectral lineshapes of spins oriented with the *g*_‖_ oriented parallel to *B*_0_ (red, left) to *g*_‖_ oriented perpendicular to *B*_0_ (blue, right) with the lineshapes of intermediate angles in shades of violet. The inset defines the angle *θ* between the interspin vector, *r*, to the externally applied field *B*_0_. The angles between the applied magnetic field *B*_0_ and the *g*_‖_ of spin A (*ϕ*_A_) and the *g*_‖_ of spin B (*ϕ*_B_) are also shown. The angles *χ*, *γ*, and *η* that describe the relative orientation of the spin pair are depicted (dashed black).

For modeling Cu(ii)-NTA at W-band we create an *in silico* sample consisting of 50 000 spin pairs. Each spin pair is comprised of two spins represented by vectors colinear with *g*_‖_, separated by an interspin vector. More details of the procedure are provided in the SI. An example of one of the repeated models that make up the *in silico* test tube is shown in Fig. 2 (inset). In red and blue are spins A and B respectively, separated by an interspin vector, *r*, in black. While each spin-pair is oriented randomly to *B*_0_, the orientation of each spin and the interspin vector is defined. The relative orientations of the two spins are described by a set of three angular parameters *χ*, *γ*, and *η*. The angle *χ* describes the angle between the *g*_‖_ axis of spin A and the interspin vector. Angles *γ* and *η* describe the polar and azimuthal angles for the *g*_‖_ axis of spin B with respect to spin A. For each spin pair, each angle is sampled from a Gaussian distribution. The standard deviation of each of these distributions is chosen to be 10° to conservatively simulate the rigidity of Cu(ii)-NTA on GB1^64,90^ although more recent work suggests that in reality these standard deviations are larger.^48^ The mean of these angles describes the average relative orientation across the generated spin pairs. Initially, the mean values of *χ*, *γ*, and *η* were chosen to be 90°, 0°, and 0°, respectively. We refer to this set collectively as relative orientations. As indicated before, the impact of relative orientations on orientational selectivity is inspected in each step of analysis by performing a grid search on many relative orientations.

After each spin pair model is oriented randomly with respect to the *B*_0_ field, the angle formed between each *g*_‖_ axis and *B*_0_, *ϕ* (*cf.*Fig. 2), is tabulated. The resonant fields for a spin at angle *ϕ* are given by:1
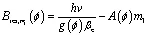
where *ν* is the microwave frequency, *h* is Planck's constant, *m*_I_ is the nuclear quantum number, and *β*_e_ is the Bohr magneton. The effective *g* and *A* values are dependent on *ϕ* and are calculated with the *g* and *A* principal axes values in eqn (2) and (3), respectively.2
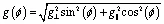
3
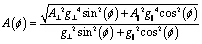


The values of *g*_‖_ and *A*_‖_ were 2.277 and 162 G, and *g*_⊥_ and *A*_⊥_ were 2.057 and 10 G, respectively.^47,91^ We calculate the lineshape for a spin by centering a Gaussian lineshape at each *B*_res_ resulting from hyperfine splitting. A Gaussian lineshape was used to account for *B*_0_ inhomogeneity. The total lineshape of a spin as a function of orientation *ϕ* is calculated with eqn (4):4
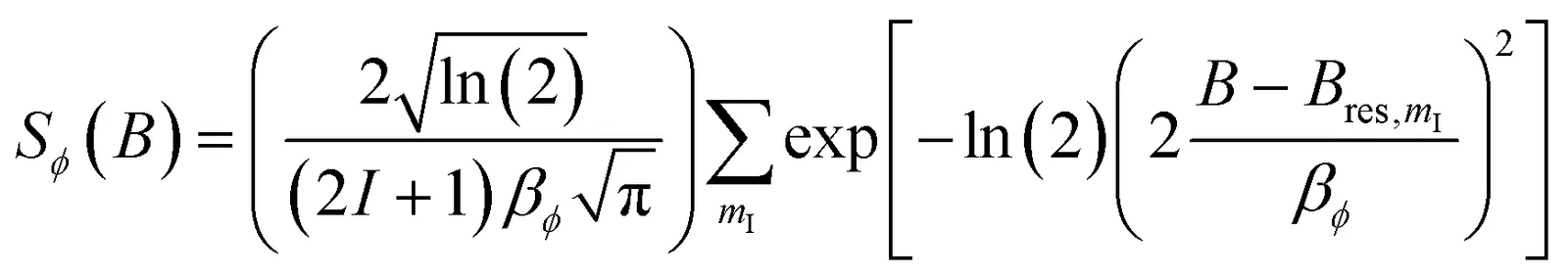
where the factor outside the summation normalizes the lineshape area, and *β*_*ϕ*_ is the full width at half maximum of the Gaussian lineshape. In previous work, the broadening parameter, *β*, was identical for all generated spins and was varied to find the best fit across the spectrum. In this work, *β*_*ϕ*_ is dependent on *ϕ* to better simulate the experimental FS-ESE. The function used to calculate *β*_*ϕ*_ as a function of *ϕ* is:5
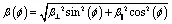


We employed the pepper module of EasySpin^92^ to simulate the experimental FS-ESE spectrum to find the optimal parameters for *β*_‖_ and *β*_⊥_*via* H-strain fitting. We then applied the optimal broadening parameters to the model to produce the simulated FS-ESE. The broadening parameters *β*_‖_ and *β*_⊥_ were found to be 400 G and 250 G. Fig. 2 shows the simulated FS-ESE spectrum based on this procedure. The lineshapes for several spins with *ϕ* that range from 0° to 90° are overlaid using a red to blue color gradient.

### Calculation of the first *ϕ*-curve

**Fig. 3:**
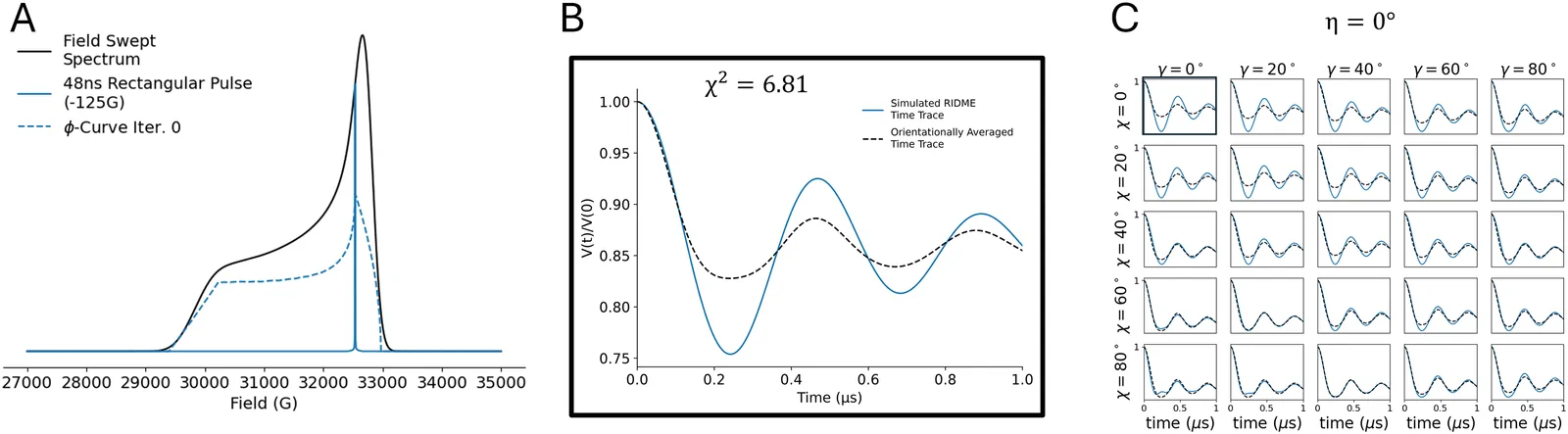
(A) The simulated FS-ESE (black) with a 48 ns rectangular pulse inversion profile (blue) centered −125 G from the maximum to simulate a RIDME experiment. (B) The normalized simulated RIDME time trace (solid, blue) overlaid on the ideal orientationally averaged signal (dashed, black). The *χ*^2^ between the RIDME time trace and the ideal time trace is 6.81. (C) A grid of simulated RIDME time traces (blue) with the orientationally averaged time trace (dashed, black) for 25 unique relative orientations defined by *χ*, *γ*, and *η* (*cf.*Fig. 2) with *η* = 0°. The upper left corner where *χ* = *γ* = *η* = 0° (bolded) denotes the position of the time trace displayed in (B).

Next, we focused on determining fields for RIDME by calculating the number of *ϕ* angles that are excited as a function of field. This curve is referred to as the *ϕ*-curve. The *ϕ*-curve was calculated by interrogating the intensity of each spin lineshape at each field. If the intensity is over the threshold limit *α*, the spin is considered excitable and contributes to the *ϕ*-curve at that field, as defined in eqn (6):

6 *X*(*B*) = {*ϕ* ∈ *U*|*S*_*ϕ*_(*B*) ≥ *α*_*ϕ*_}

where *X*(*B*) is the set of spins at field *B*, *U* represents all values of *ϕ*, and *S*_*ϕ*_(*B*) is the lineshape of a spin with orientation *ϕ* at field *B*. The limiting parameter α was set similar to previous work,^80^ where *α* was optimized for peak normalized Lorentzian curves with a width of 40 G at half maximum. However, in this work, we model spin lineshapes with area normalized Gaussian curves. Additionally, the broadening parameter, which informs the optimal choice of *α*, is now dependent on spin orientation *ϕ*. Therefore, in this work, each spin lineshape is interrogated with a unique limiting parameter *α*_*ϕ*_. The limiting parameter ranges so that the probability mass of the spin lineshape above *α*_*ϕ*_ is consistent with that of previous work. Specifically, *α*_*ϕ*_ ranges from 4.32 × 10^−4^ for *ϕ* = 0° to 6.92 × 10^−4^ for *ϕ* = 90°. Varying the limiting parameter values ensures that the weighting of each line is not biased by the total intensity. These limiting parameters are represented graphically in Fig. S1 of the SI. Fig. 3A shows the simulated FS-ESE in black overlaid on *ϕ*-curve 0 as a blue dashed line. For Cu(ii)-NTA at W-Band, the maximum of *ϕ*-curve 0 is −125 G from the maximum of the FS-ESE (*cf.*Fig. 3A). The shift of *ϕ*-curve maximum away from the canonical *g*_⊥_ position is due to the overlap of spectral lineshapes with *ϕ* in the range 70°–90° (*cf.*Fig. 2, blue). A RIDME at this field thus enhances the excitation of different *θ* angles.

### Calculation of RIDME

Next, we simulate RIDME performed at −125 G from the max field position. To simulate RIDME, we calculate the dipolar frequency for each of the excited spin pairs with eqn (7):7
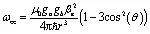


We use the dipolar frequency to calculate the time domain signal for each spin pair with eqn (8):

8 *V*_*n*_(*t*) = *P*_Flip_cos(*ω*_ee_*t*)

where *P*_Flip_ is the probability that a given spin's partner will flip in the mixing time *T*_mix_, given by eqn (9):^93^9
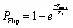
where *T*_1_ is the longitudinal relaxation time. For this model, *T*_1_ was assumed to be independent of spin orientation and set to 300 µs for all spins. *T*_mix_ was set to 100 µs. The choice of *T*_1_ and *T*_mix_ only affects the modulation depth of the simulated RIDME signal, but not the frequency of modulation or the identity of magnetic fields.

We then take the weighted sum of the resulting time domain signals with eqn (10):10
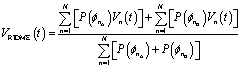
where *N* is the total number of spin pair models. The denominator of eqn (10) ensures the signal is normalized to 1 at *t* = 0. *P*(*ϕ*_*n*_A__) is the probability of excitation for spin A of the *n*^th^ spin pair by a π/2 RIDME pulse. *P*(*ϕ*_*n*_B__) is the analogous probability for the B spin on the spin pair. The probability of excitation for a spin with orientation *ϕ* is given in eqn (11):11
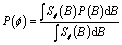
where *P*(*B*) is the pulse inversion profile of the pulses in the echo detection sequence. This probability is needed to identify excited spins and their orientations, and to properly scale each spin pair's oscillating signal to the total simulated RIDME time trace. Note that the probability of the partner spin flipping is determined stochastically using eqn (9). For this model, a 48 ns rectangular pulse was used to replicate experimental parameters. Fig. 3A shows the simulated FS-ESE in black. The 48 ns pulse inversion profile is shown in blue, centered at −125 G from the maximum of the simulated FS-ESE. The orientationally averaged signal is the sum of all the simulated time domain signals from each spin-pair, *i.e.*, *P*(*ϕ*_*n*_A__) = *P*(*ϕ*_*n*_B__) = 1.

### Quantification of orientational selectivity

To quantify the extent of orientational selectivity for each acquisition scheme, we calculate the *χ*^2^ value between the simulated RIDME and orientationally averaged time domain signals, described by eqn (12):12
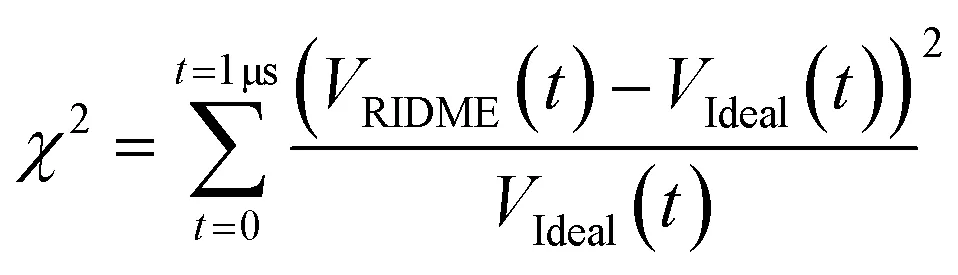
where *V*_Ideal_(*t*) is the orientationally averaged signal, adjusted to match the modulation depth of the simulated RIDME signal. Eqn (13) describes how the modulation depth adjustment is conducted:

13 *V*_ideal_(*t*) = *V*_total_ × *λ* + (1 − *λ*)

where *λ* is the modulation depth of the simulated RIDME signal.

Fig. 3B presents the ideal signal (sum of all spins) with a black dashed line overlaid on the simulated RIDME time trace in blue. The resulting *χ*^2^ value between these two traces is 6.81. Visually, the simulated RIDME time trace modulates at a similar frequency to the ideal case but has a reduced damping of oscillations. The reduced oscillation damping of the simulated RIDME signal suggests orientational selectivity, specifically an oversampling of the *θ* = 90° case. For this relative orientation, where *χ* = *γ* = *η* = 0°, both the *g*_‖_ and the interspin vector are aligned. Thus, by primarily exciting spins where *ϕ*_A_ ≈ *ϕ*_B_ ≈ 90°, an oversampling *θ* = 90° is expected. Note that the extent and form of orientational selectivity will depend on the relative orientation of the spin pair and the distribution in angles. To analyze the efficacy of an acquisition scheme, we simulate RIDME on *in silico* samples with 125 different relative orientations. These 125 relative orientations are generated by iterating the mean values for *χ*, *γ*, and *η* through five equally spaced values: 0°, 20°, 40°, 60°, and 80°. A step size of 20° was selected based on the 10° standard deviation of each Gaussian curve, ensuring overlap between sets of relative orientations. Fig. 3C presents the twenty-five time-domain plots with *η* = 0°, a subset of the 125 relative orientations tested. Each plot corresponds to a unique pairing of *γ* and *χ*. The other subsets where *η* > 0° are shown in Fig. S2–S5 of the SI. Although an orientationally averaged signal is achievable for a small subset of relative orientations (*cf.* Fig. S7 and Fig. 3C) the use of only one field would be reliable only if the relative orientation is known *a priori*.

### Generation of additional field positions for acquisition

**Fig. 4:**
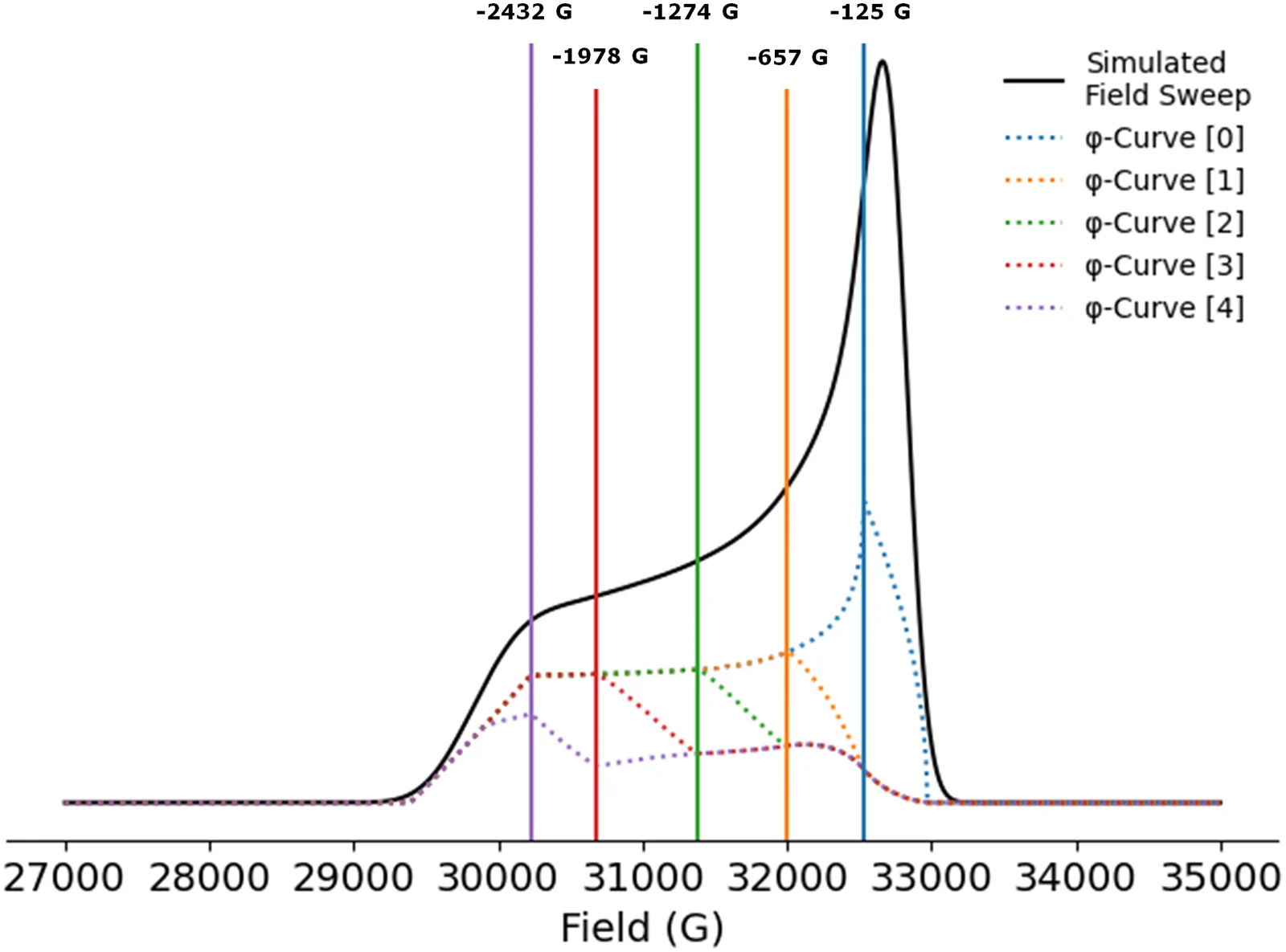
The simulated FS-ESE spectrum (black) with five iterations of the *ϕ*-curve in blue, gold, green, red, and violet respectively. The field position of the maximum of each curve is marked with a vertical line in the same color. The maxima with respect to the maximum of the simulated FS-ESE are −125 G, −657 G, −1274 G, −1978 G, and −2432 G respectively.

Thus, as per our workflow from Fig. 1, we find additional field positions to perform RIDME. To this end, we remove the spin pairs excited by the first simulated RIDME and recalculate the *ϕ*-curve. Fig. 4 portrays the recalculated curve, *ϕ*-curve 1, in gold. The maximum of this position is −657 G from the maximum of the FS-ESE. This cycle of removing excited spin pairs and finding additional fields continues until five total field positions are identified. These are −125 G, −657 G, −1274 G, −1978 G, and −2432 G from the maximum of the spectrum (*cf.*Fig. 4). Because the recalculation of the *ϕ*-curves excludes the previously excited spin pairs, the exact field positions may be dependent on the relative orientation of the spin pair. Thus, the five iterations of the *ϕ*-curves for a grid of unique relative orientations are presented in Fig. S6 of the SI. Notably, the field positions are remarkably consistent until the third iteration where slight deviations can be seen.

### Combinations of field positions provide an optimum acquisition scheme

To decipher the optimum acquisition scheme, we tested every combination of these five positions. First, we summed the RIDME from any two acquisitions. There are 10 possible two-field combinations. We calculated the *χ*^2^ values for a grid of 125 relative orientations. For two fields the best range was obtained by summing acquisitions at −125 G and −657 G from the spectral maximum.

**Fig. 5:**
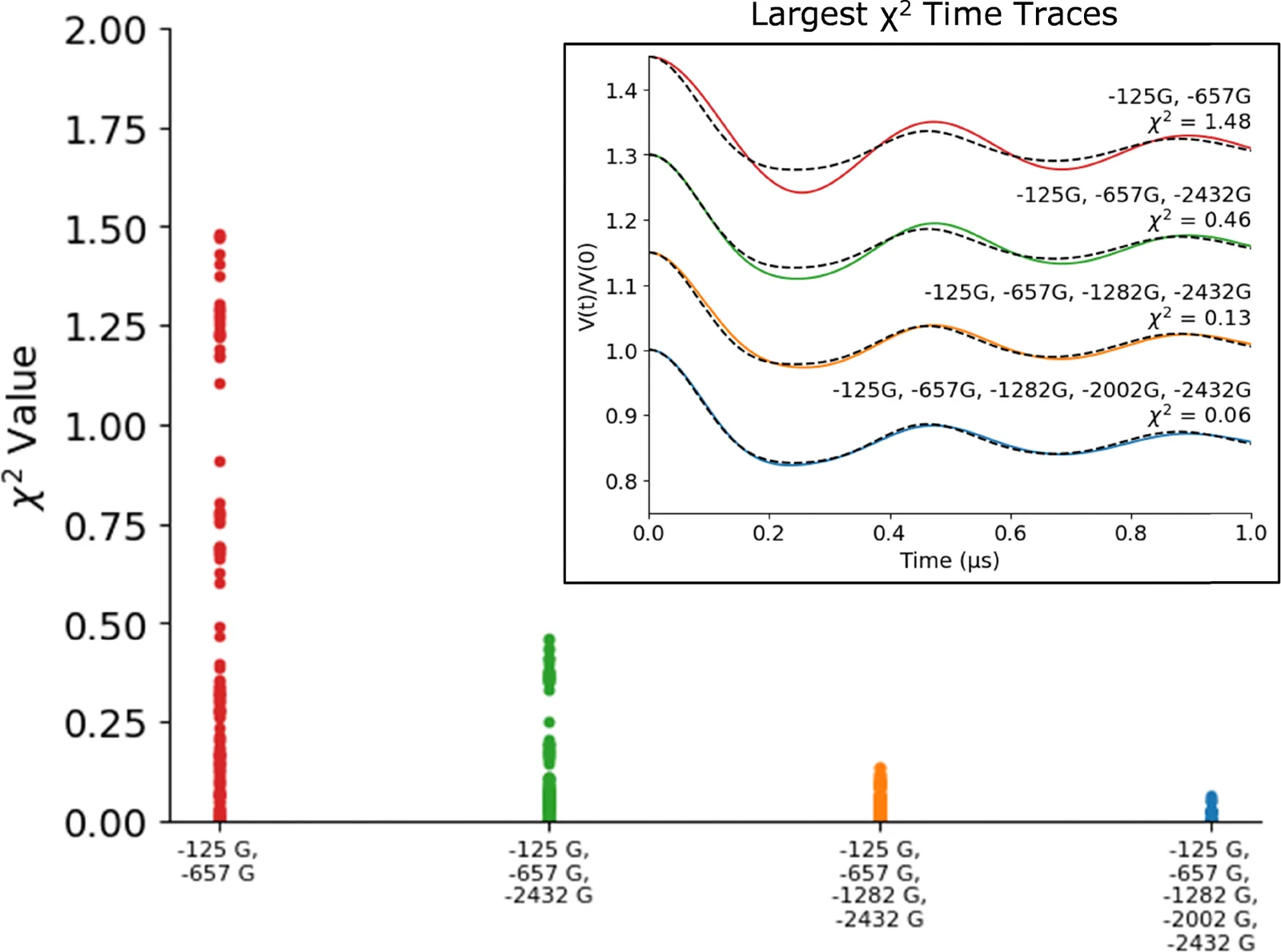
A scatter plot of *χ*^2^ values for the 2-field (red), 3-field (green), 4-field (gold), and 5-field (blue) position acquisition schemes applied to a systematic sampling of 125 unique relative orientations. The acquisition schemes presented are those with the smallest range of *χ*^2^ values in their respective categories. The signals that give the largest *χ*^2^ value from each acquisition scheme, overlaid on the ideal time domain signal (dashed, black) are shown in the inset.

Fig. 5 shows the range of *χ*^2^ for this two-field acquisition scheme. The *χ*^2^ for each relative orientation is show in red. The data for all ten combinations of two field positions is shown in Fig. S8 of the SI. The *x*-axis in Fig. 5 identifies the field values of these combinations. Similarly, the acquisition schemes with the smallest range of *χ*^2^ values for combinations of three and four field position RIDME are plotted in green and gold, respectively. The range of *χ*^2^ values obtained by summing all five field positions is shown in blue. The *χ*^2^ values for every possible 3- and 4-field combination of the five field positions are shown in Fig. S9 and S10 in the SI.

To contextualize the range of *χ*^2^ values, the time domain data for the highest *χ*^2^ value for each acquisition scheme is plotted in the inset of Fig. 5. For each trace, the data for an ideal RIDME is overlaid using a dashed black line. The inset shows that a *χ*^2^ value of 1.48, the largest for the two-field acquisition scheme, visibly deviates from the ideal time trace. Similarly, a *χ*^2^ value of 0.46, the largest value for the three-field acquisition scheme, also deviates from the ideal. However, for the four field and five field acquisition schemes, with maximum *χ*^2^ values of 0.13 and 0.06 respectively, differ very little from the ideal. The best four-field acquisition consists of summing RIDME data acquired at −125 G, −657 G, −1282 G, and −2432 G from the field of maximum intensity. The additional collection at the fifth field position leads to minor improvements.

**Fig. 6:**
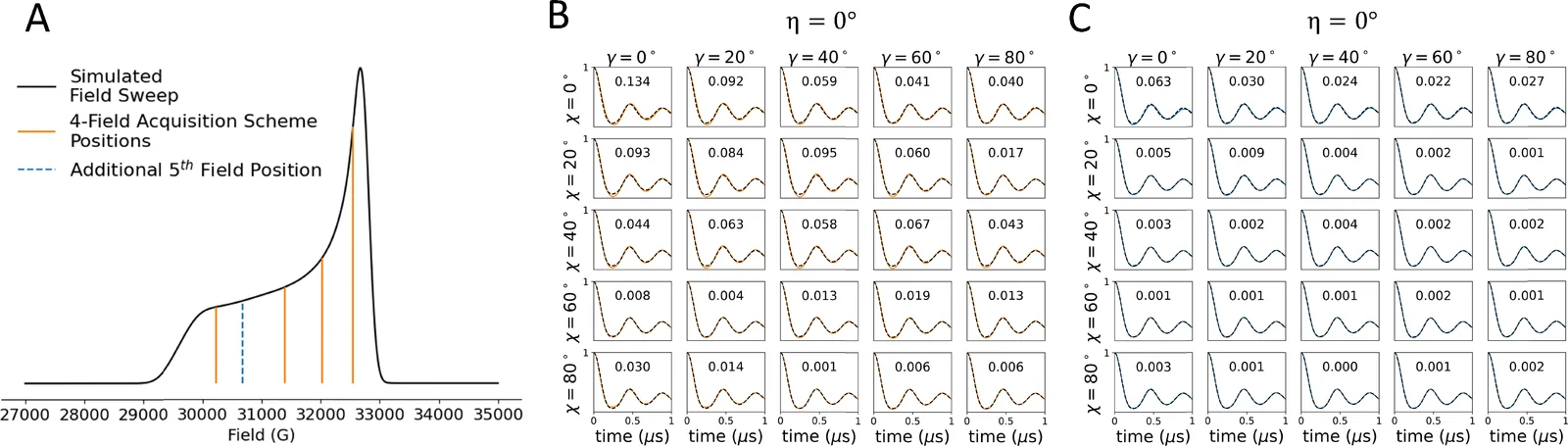
(A) The field positions which make the optimal 4-field acquisition scheme (gold), centered −125 G, −657 G, −1282 G, and −2432 G from the maximum of the simulated FS-ESE (black). The additional field position for the 5-field acquisition scheme (dashed blue) is centered −2002 G from the maximum of the simulated FS-ESE. (B) The resulting time traces of the optimal 4-field acquisition scheme applied to a grid of unique relative orientations. Each time domain plot is overlaid on the ideal time trace (black, dashed) and the resulting *χ*^2^ value is displayed. (C) The analogous time traces using the optimal 5-field acquisition scheme.

Fig. 6 elaborates on the difference between the four- and the five-field acquisition scheme. Fig. 6A illustrates the positions of the simulated RIDMEs on the FS-ESE. The time traces for a grid of different relative orientations, analogous to those shown in Fig. 3C, are shown for the four-field acquisition scheme in Fig. 6B. The same plots are shown for the five-field acquisition scheme in Fig. 6C. Notably, the four field acquisition scheme adequately averages the time domain data sufficiently regardless of the relative orientation.

### Key considerations for identified field positions

The first position of the *ϕ*-curve depends only on the *g*- and hf-tensors of the spin. Although the *g*- and hf- tensors can vary slightly depending on the properties of the spin-labeling site, these variations are small for dHis-Cu(ii).^94^ Thus the first position is relatively conserved for this label. In our procedure the subsequent *ϕ*-curves and therefore fields are identified by removing all spins that contribute to RIDME at the previous field. Thus, these field positions can, in principle, depend on three factors.

The first factor is the orientational flexibility of the simulated spin pair. Herein, we have used a standard deviation of 10° for all angles. This value is relatively conservative based on estimates from earlier MD simulations and quantum mechanical calculations.^80^ In addition, experimental data suggests that Cu(ii)-NTA bound to α-helices may have orientational flexibility as high as 28°, 72°, and 6° for the standard deviations of the *χ*, *η*, and *γ*, angles respectively.^48^ For the Cu(ii)-NTA label this flexibility arises from the elastic coordination of the label to the dHis sidechains. The large range of bond lengths and bond angles leads to distribution in the direction of the *g*_‖_ axis, and the role of this orientational flexibility in mitigating orientation effects in DEER have been reviewed before.^37,90,95^ Due to the large range of relative orientations dHis-Cu(ii) based distances are generally non-orientationally selective at X-band,^90^ and only two/three acquisitions are needed at Q-band.^79,80^ In fact, the use of alternative Cu(ii) labels that marginally reduce the *g*-anisotropy is sufficient to wash out orientational effects at Q-band.^96^ On the other hand, Cu(ii) systems that exhibit much tighter coordination can exhibit orientational selectivity in PDS measurements even at X-band.^97–100^ Such systems would require more field positions that can be determined using appropriate parameters for orientational flexibility.^45^

Second, the broadening parameters used will dramatically affect the range of fields at which a spin is sampled. Thus, it is critical to calibrate the broadening parameters by simulating the FS-ESE spectrum. Lastly, we consider the bandwidth of the pulses used to simulate RIDME. The 48 ns pulse used here is achievable at W-Band even with commercial hardware.^56,74,85,101,102^ Nonetheless, systems limited to pulses longer than 48 ns may require additional field positions to achieve an orientationally averaged signal. Inversely, instrumentation with wider resonator bandwidths have recently been developed,^103,104^ which can potentially reduce the number of field positions needed to achieve an orientationally averaged spectrum. We have shown in previous work that an increase in frequency offset for the DEER experiment reduced the necessary number of fields from three to two for Cu(ii)-NTA at Q-Band.^79^

## Experimental results

### Protein samples

RIDME was performed on two GB1 mutants. The RIDME experiment was chosen to avoid the losses from the reduced modulation depth with DEER, especially at the lower magnetic fields, which compromise sensitivity. The GB1 mutants were E15H/T17H/K28H/Q32H and I6H/N8H/K28H/Q32H. Each mutant possesses two dHis sites. Both mutants contain the K28H/Q32H mutation on the α-helix of GB1. The mutants differ in the position of the second dHis site. The I6H/N8H mutation occurs on the first β-sheet of GB1, whereas the E15H/T17H mutation occurs on the third β-sheet of GB1. By labelling different β-sheets, we can test the optimal acquisition scheme on two different relative orientations of spin pairs.

**Fig. 7:**
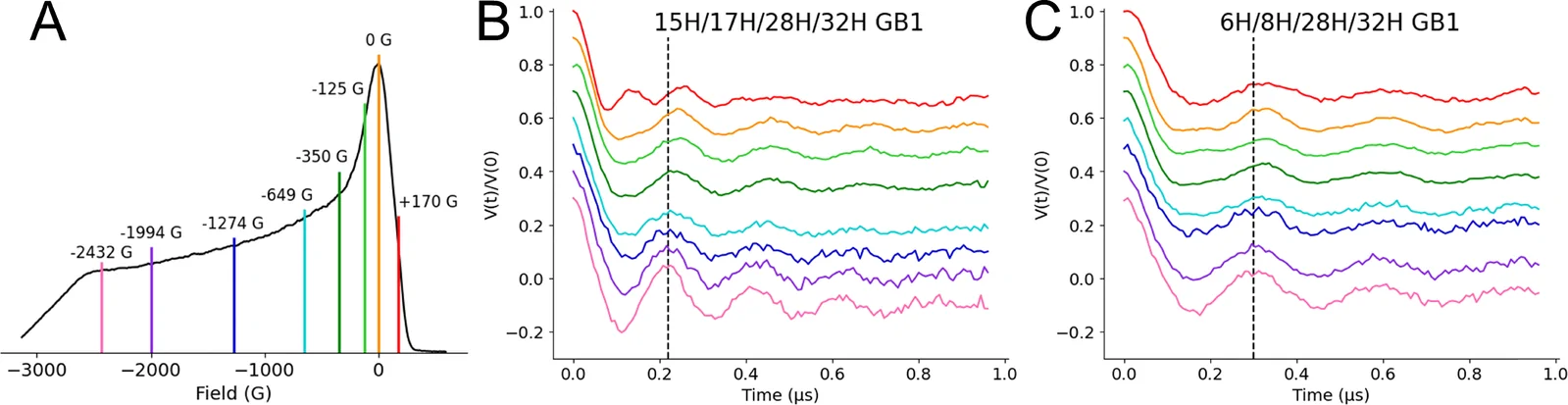
(A) An experimental FS-ESE collected with the HiPER spectrometer (black). Overlaid are the eight field positions, −2432 G (pink), −1994 G (violet), −1274 G (blue), −649 G (cyan), −350 G (dark green), −125 G (light green), 0 G (orange), and +170 G (red) where RIDME was conducted. (B) The background subtracted and normalized RIDME time traces on the I6H/N8H/K28H/Q32H GB1 mutant. The time traces are offset for visibility and go from high field to low field top down, maintaining the color scheme presented in A. (C) The analogous RIDME data for the E15H/T17H/K28H/Q32H GB1 mutant. The vertical dashed lines in the data are provided to help visualization of differences.

RIDME data was collected at eight field positions across the FS-ESE spectrum, as shown in Fig. 7A. Fig. 7B shows the background corrected RIDME signals collected at each field for 15H/17H/28H/32H GB1. The frequency domain Pake patterns for each time trace are shown in Fig. S11 of the SI. The high field time trace, in red, oscillates at a higher frequency than any other field collected. A vertical dashed line is overlaid on Fig. 7B to illustrate the shift in frequency between the RIDME time traces.

Fig. 7C shows the RIDME time domain data at the same field positions for the 6H/8H/28H/32H GB1, with the Pake patterns in Fig. S11 of the SI. Similarly, a vertical dashed line has been overlaid to illustrate the shift in frequency. The change in oscillation frequency between field positions is indicative of orientational selectivity. Note also that the time traces are distinct for the two samples at a given field position. This is most evident in the red traces shown in Fig. 7B*versus*Fig. 7C. This difference highlights the effects of the relative orientation of the *g*-tensors on PDS data.

Both data sets can be analyzed to extract information on the relative orientations of the spin centers. Such analyses have been widely applied to biomolecules containing nitroxide labels or radical cofactors, where orientation-selective distance measurements provide additional structural constraints that help define protein conformations, conformational changes, and dynamic modes.^53,105–110^ For Cu(ii) centers, however, orientation-selective experiments, while possible, are more challenging.^48,111–113^ The broad and highly anisotropic Cu(ii) EPR spectrum requires data collection over a wide field range, and acquisition at magnetic fields below the *g*_⊥_ region is often particularly time-consuming due to reduced sensitivity. Consequently, obtaining sufficient orientation-selective information can become experimentally demanding, especially for systems with limited signal intensity. In cases where only distance constraints are required, the additional experimental effort associated with orientation selection may outweigh its benefits, making optimization of the data collection strategy essential.

### Application of acquisition schemes

We apply the suggested acquisition scheme by taking the weighted average of the RIDME time traces. Each raw time trace closest in field position to the fields suggested by the model is normalized such that the zero-time is one and then weighted to the intensity of the FS-ESE at that field.^48^ This weighted average is used to account for the population differences at each field position. Alternatively, the integrated RIDME echo at each position may be used. The two procedures are identical as long as relaxation anisotropy is small. The weighted averages for each acquisition scheme are shown in Fig. S12. Each signal is analyzed with a modified Tikhonov regularization^89^ in DeerAnalysis 2022,^88^ and with DEERNet,^114^ shown in Fig. S13 and S14.

**Fig. 8:**
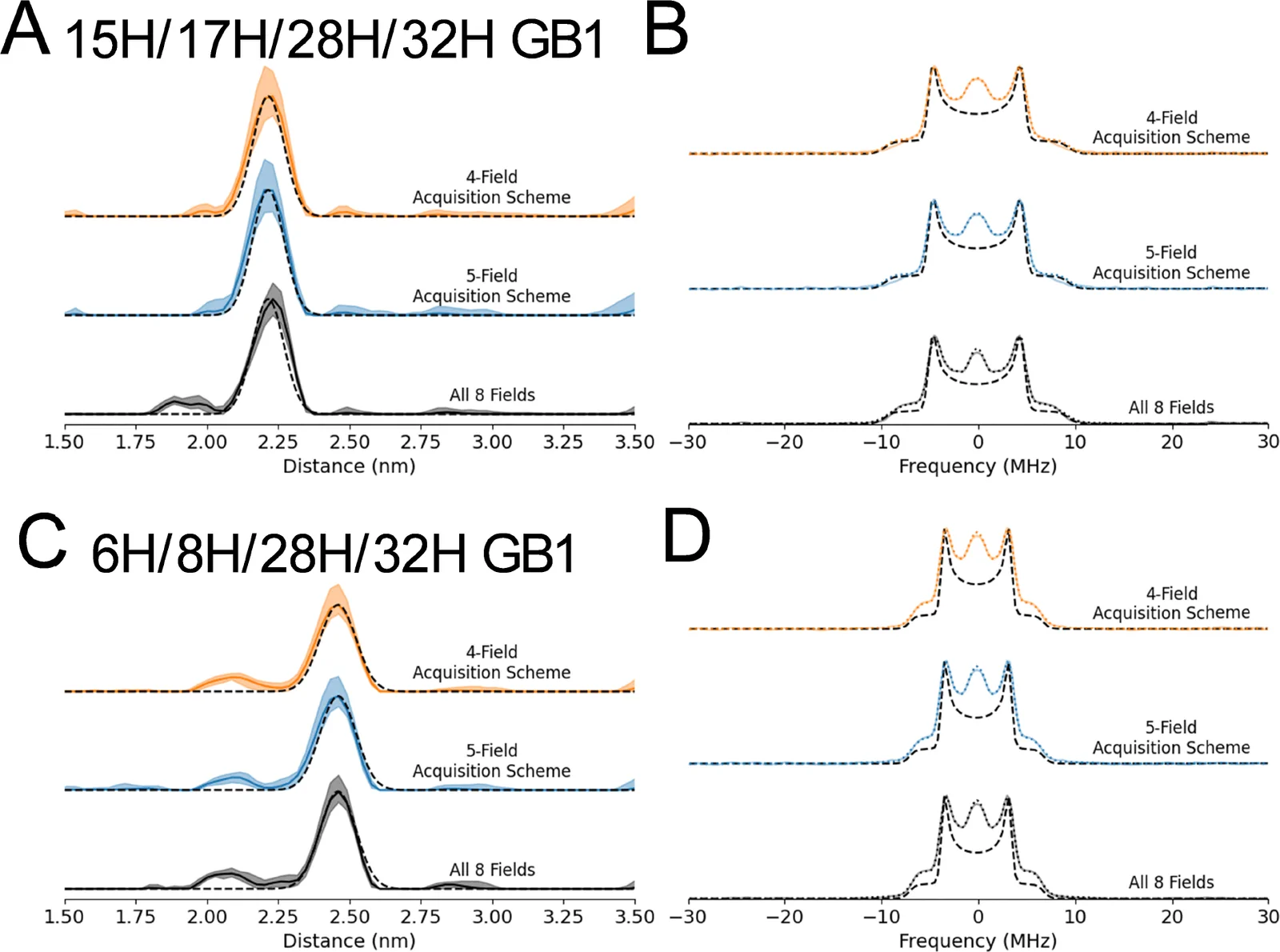
(A) The distance distributions from the weighted average time domain signals for the optimal 4-field (gold) and 5-field (blue) and 8-field (black) acquisition schemes collected on the E15H/T17H/K28H/Q32H GB1 mutant. A Gaussian distribution is shown as dashed black line. Each distance distribution has been vertically offset for visibility. The shaded area is the 95% confidence interval calculated by varying the background subtracted component. (B) The Pake patterns corresponding to each of the acquisition schemes presented in A. The Pake pattern corresponding to the ideal Gaussian distance distribution is plotted in a dashed black line. (C) The analogous distance distributions for the I6H/N8H/K28H/Q32H GB1 mutant. (D) The corresponding Pake patterns for the acquisition schemes presented in C.

Fig. 8A shows the distance distributions obtained using the 4-field and the 5-field schemes for the 15H/17H/28H/32H mutant. Both schemes produce the expected most probable distance of 22 Å for the 15H/17H/28H/32H mutant (top two distributions).^36^ The distance distributions are also identical within experimental and analysis errors. On the other hand, the distribution obtained by summing all eight acquisitions shows distinct differences, namely a peak around 18 Å (*cf.* black distribution). The plot also shows the fit of the main distance peak to a Gaussian distribution (*cf.* dashed black line). From this Gaussian we use a dipolar kernel from DeerLab^115^ to calculate the expected Pake pattern (*cf.* dashed black line Fig. 8B). We propose that the 18 Å peak for the 8-field acquisition scheme originates from oversampling of *θ* = 0°. This interpretation is supported by an examination of the Pake patterns shown in Fig. 8B with respect to the Pake pattern for the Gaussian. The 4 and 5 field acquisitions yield similar Pake patterns both to each other, and to the calculated Pake pattern. On the other hand, there is a noticeable increase at 8 MHz for the 8-field acquisition scheme, which corresponds to the *θ* = 0° orientations. This data also illustrates the value of proper orientational averaging in PDS measurements. The extraction of distance distributions from non-statistically sampled data can lead to artificial peaks in the distribution, narrowing of the distribution, or changes in the mean distances. Comparisons of the distance distributions between the 4-field acquisition scheme and individual fields for the 15H/17H/28H/32H mutant, shown in Fig. S15, illustrate these effects. The artificial narrowing of the main distance, as well as the bimodality encountered when *θ* = 0° is oversampled, are clearly visible in the single field experiments.

Fig. 8C and D show the analogous results for the 6H/8H/28H/32H GB1 mutant. Each acquisition scheme yields the expected most probable distance of 24 Å for this mutant.^36^ However, the 6H/8H/28H/32H mutant produced minor peaks at 21 Å in all tested acquisition schemes, suggesting residual orientational selectivity. The persistent orientational selectivity is not entirely unexpected. Previously, we have shown that average orientation angles for this mutant are (*χ* = 100.0°, *γ* = 97.5°, and *η* = 101.6°).^90^ Thus, the *g*_‖_ of spin A, the *g*_‖_ of spin B, and the interspin vector are all nearly perpendicular. In this relative orientation, RIDME near the *g*_⊥_ region will sample both *θ* = 90° and *θ* = 0°. Indeed, this feature can be directly observed in the Pake pattern for this mutant (*cf.* second line of Fig. S11D in SI). Due to this, the correct statistical sampling is challenging for this relative orientation.

Nevertheless, our computational results indicate that the combination of 4 fields should largely suppress orientational selectivity, even for the most difficult of relative orientations (*cf.*Fig. 5 inset). The discrepancy can be due to *B*_1_ inhomogeneity inherent to the HiPER spectrometer. In the computational methodology two spin-pairs that have the same relative orientation but are located in different regions of the sample would both contribute to the RIDME signals (albeit with different weights). In experiments, the pulse imperfections can lead to differential propagation through the RIDME coherence pathway for the spins. Further, the acquisition scheme is particularly sensitive to the placement of the −125 G position. Each relative orientation produces a peak at this point (*cf.* Fig. S6 in the SI). Thus, misalignment, especially toward the maximum of the FS-ESE, may be the cause of oversampling *θ* = 0° for mutually perpendicular spin pairs.

Despite the residual orientational selectivity of the 6H/8H/28H/32H GB1 mutant, the 4-field acquisition schemes yield similar information to the 8-field acquisition schemes. The data collection times are ∼28 h, ∼23 hours, and ∼16 hours for the 8-field, 5-field, and 4-field schemes, respectively. If information on relative orientations is not desired, the optimized 4-field position acquisition scheme yields quality distance results in a shorter amount of time.

## Conclusions

We conceptually analyze the acquisition scheme to achieve orientationally averaged RIDME data on dHis-Cu(ii) at W-band. We show that orientationally averaged RIDME data can be acquired in as few as four or five experiments. These field positions are −125 G, −657 G, −1282 G, and −2432 G lower than the field of maximum intensity. These field positions depend in part on the pulse lengths, the broadening parameters and the orientational flexibility of the labels. These results are notable because they suggest that collection at these fields can effectively excite a sub-ensemble of spins that recapitulates statistical excitation. Notably, the scheme involves excitation of only ∼0.43% of spins.

To verify the *in silico* results, we applied the acquisition scheme experimentally on the HiPER spectrometer. We conducted W-band RIDME on two different mutants of the GB1 protein with commonly accessible 48 ns pulses. For each mutant, we found that the proposed four-field acquisition scheme performed as well or better than an eight-field acquisition scheme. In fact, the eight-field acquisition scheme may result in oversampling of particular orientations, resulting in artifacts in the distance distribution.^48^ However, the degree of this oversampling will be sensitive to the particular spin pair relative orientation, and thus the labeling sites of Cu(ii)-NTA.

We anticipate that this conceptual model will facilitate acquisition schemes for distance measurements using other pulsed EPR techniques and rigid spin labels.^61,62,96,116^ Ultimately, we expect this process to facilitate the merger of rigid spin labels and high frequency pulsed EPR.

## Conflicts of interest

There are no conflicts to declare.

## Supplementary Material

CP-028-D6CP02541B-s001

## Data Availability

The research data underpinning this publication will be accessible at DOI: https://doi.org/10.17630/2d6ea446-f968-4146-8f1f-76379b1b68e2.^117^ Supplementary information: details of computational analysis, primary experimental data and analysis are provided in SI. See DOI: https://doi.org/10.1039/d6cp02541b.
