# Supplementary material for: dHis-Cu(ii) based EPR distances at W-band: navigating the challenges of excitation
Source: Phys Chem Chem Phys. 2026 Sep 14;28(37):22657–68. doi: 10.1039/d6cp02541b (PMC13572940; doi:10.1039/d6cp02541b)
Supplement: Supplementary file 1 [file CP-028-D6CP02541B-s001.pdf]

Supplementary Information (SI) for Physical Chemistry Chemical Physics.
This journal is © the Owner Societies 2026

Generation and Rotation of Model Spin Pairs:

The spin pairs are generated with the 'numpy' python library as three unit-vectors representing the $g_{\parallel}$ of each spin, and the interspin vector. The interspin vector and $g_{\parallel}$ of spin B are rotated in accordance with the relative orientation parameters and the distributions widths. This rotation is done by applying a rotation matrix via the 'Rotation' class in the 'scipy' python library. The properly oriented spin pair is then randomly oriented as a unit, i.e. all three vectors are rotated, using the same function but with a rotation matrix generated via the "Rotation.random()" function. We varied the number of spin pairs from 1000 to 1 million and tested the procedure for one relative orientation. We obtained the same results for 10,000-1 million spin pairs. A value of 50,000 was chosen to ensure robust results without sacrificing computer resources.

Limiting Parameter Values

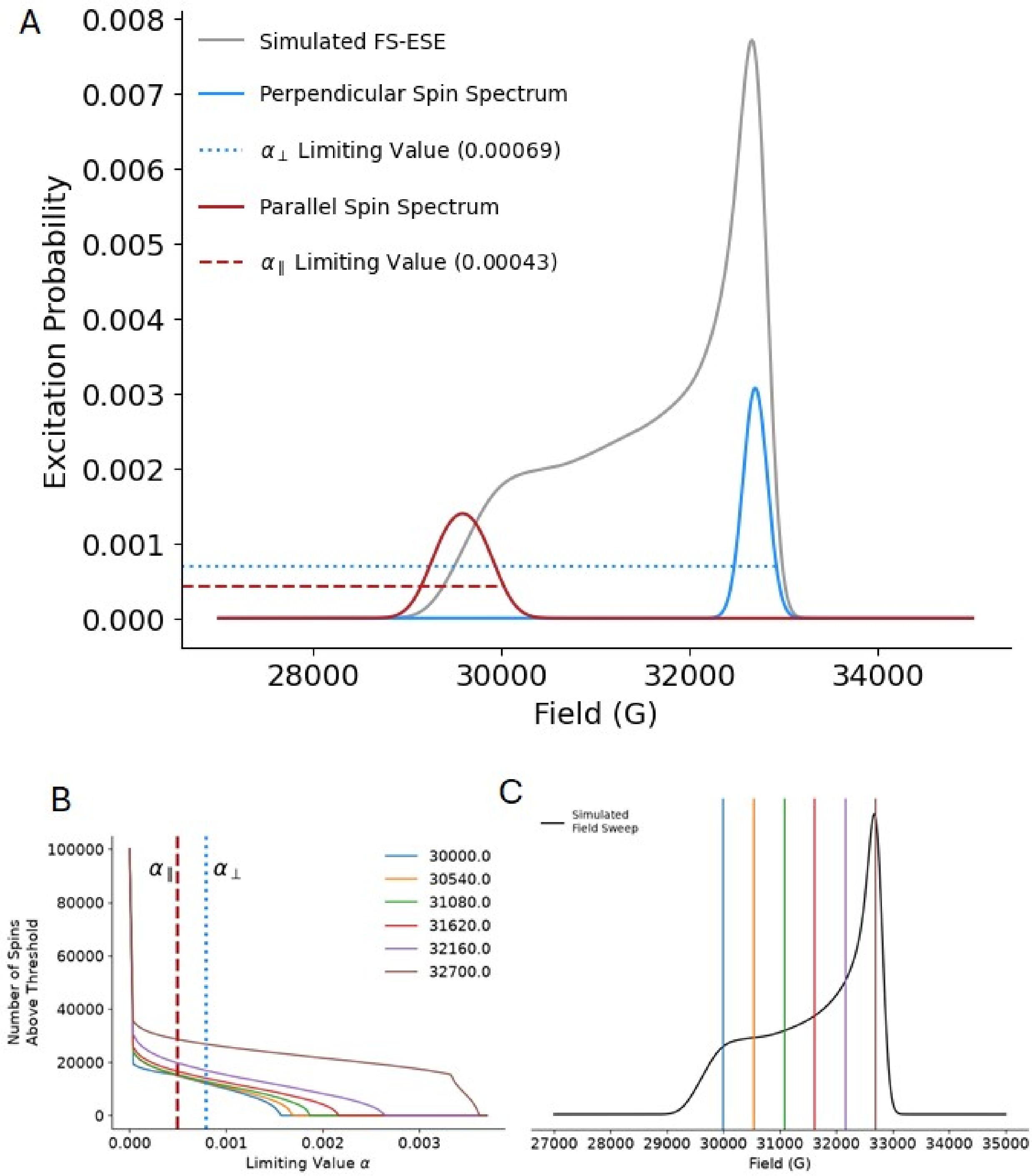

Figure S1: *A) The simulated spectra for Cu(II)-NTA spins with* $g_{/\!/}$ *oriented parallel (φ=0°, red) and perpendicular (φ=90°, blue). The red dashed and blue dotted lines depict the limiting parameter values used to calculate the φ-curves. The simulated Cu(II)-NTA FS-ESE (grey) is overlaid. B) The visualization of the limiting parameters for the parallel region (red dashed) and the perpendicular region (blue dotted) overlaid on the plot of the number of spin pairs above the limiting value threshold as a function of the limiting parameter at six fields across the FS-ESE. C) The visualization of the six fields plotted in B).*

Single Field Simulated RIDME on Relative Orientation Sets

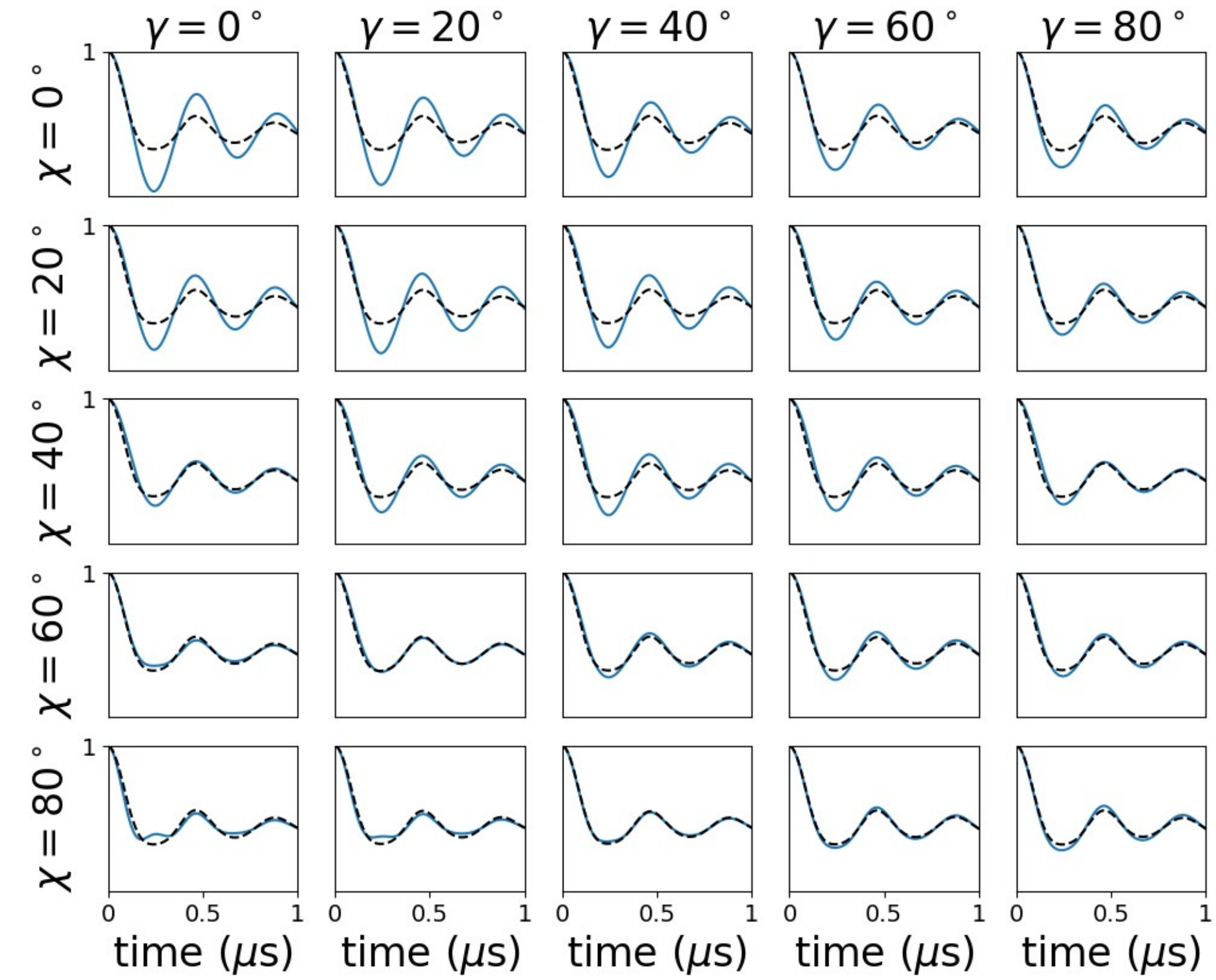

Figure S2: *The simulated and ideal RIDME time traces were obtained for different relative orientations defined with values of χ and γ angles. The value of η is 20°. The standard deviation for each angle was set at 10°. The simulated RIDME was obtained at a field of -125 G from the maximum of the FS-ESE which corresponds to the maximum of the φ-curve as discussed in main text (cf. Figure 3A in the main text for more details).*

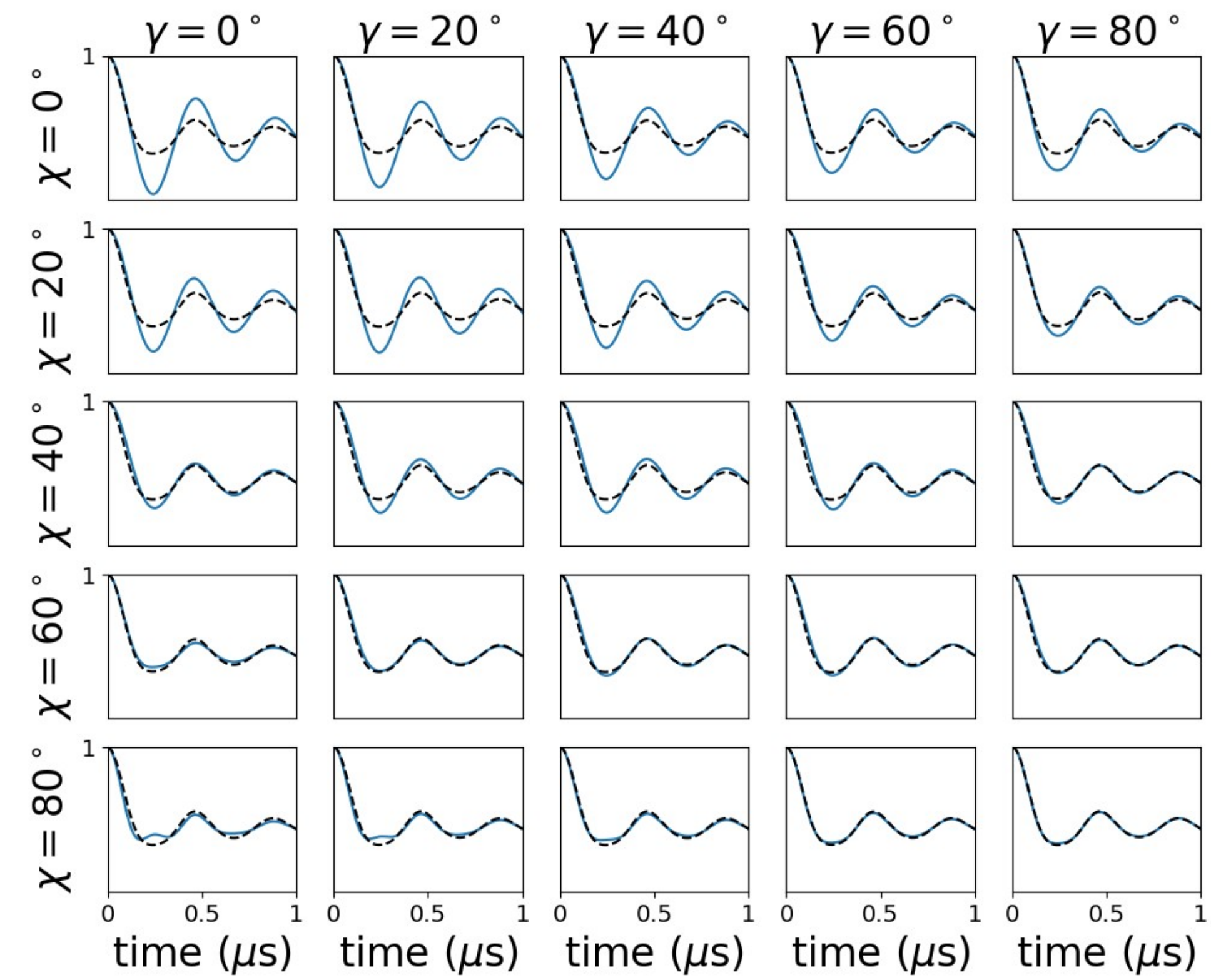

Figure S3: *The simulated and ideal RIDME time traces were obtained for different relative orientations defined with values of χ and γ angles. The value of η is 40°. The standard deviation for each angle was set at 10°. The simulated RIDME was obtained at a field of -125 G from the maximum of the FS-ESE which corresponds to the maximum of the φ-curve as discussed in main text (cf. Figure 3A in the main text for more details).*

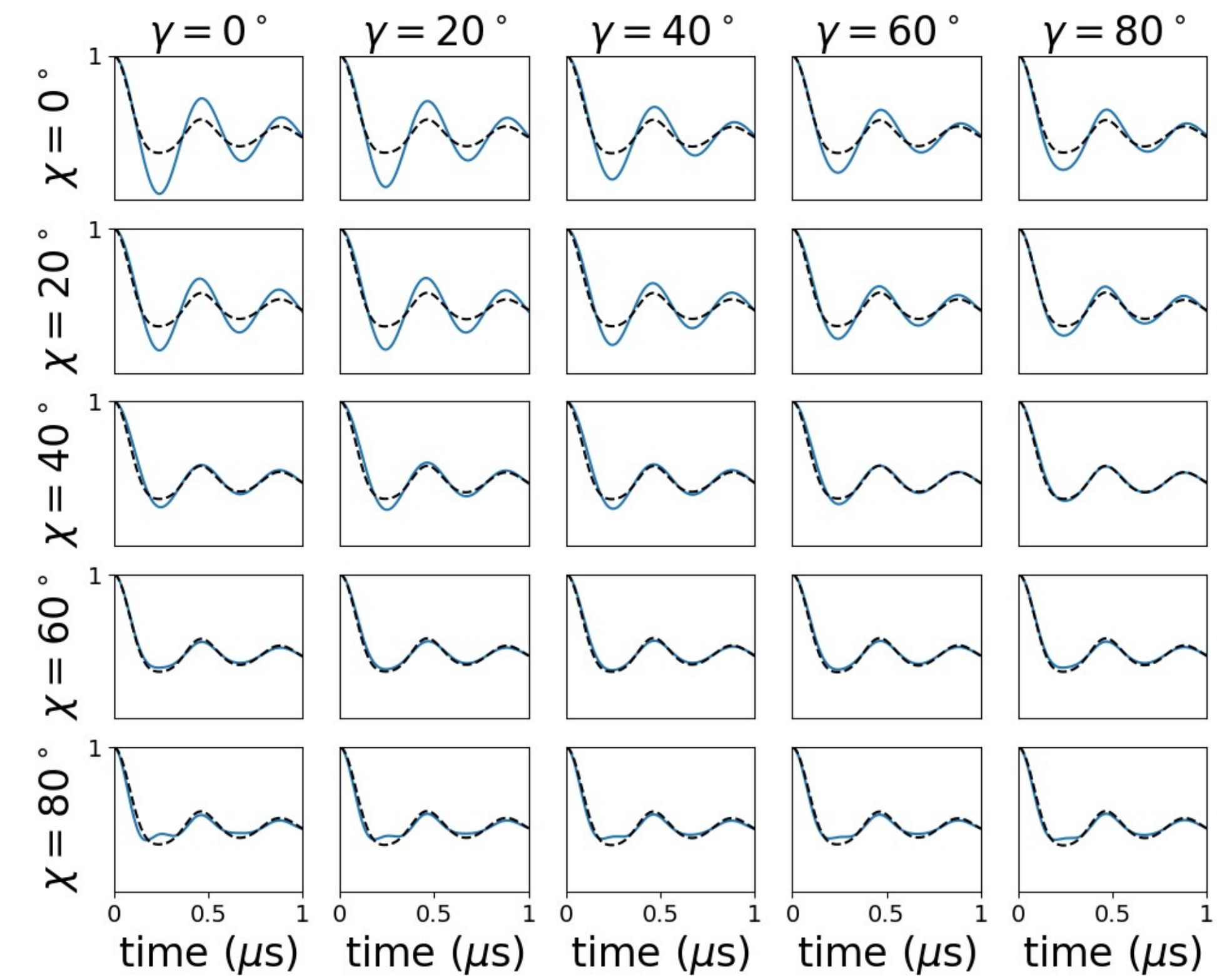

Figure S4: *The simulated and ideal RIDME time traces were obtained for different relative orientations defined with values of χ and γ angles. The value of η is 60°. The standard deviation for each angle was set at 10°. The simulated RIDME was obtained at a field of -125 G from the maximum of the FS-ESE which corresponds to the maximum of the φ-curve as discussed in main text (cf. Figure 3A in the main text for more details).*

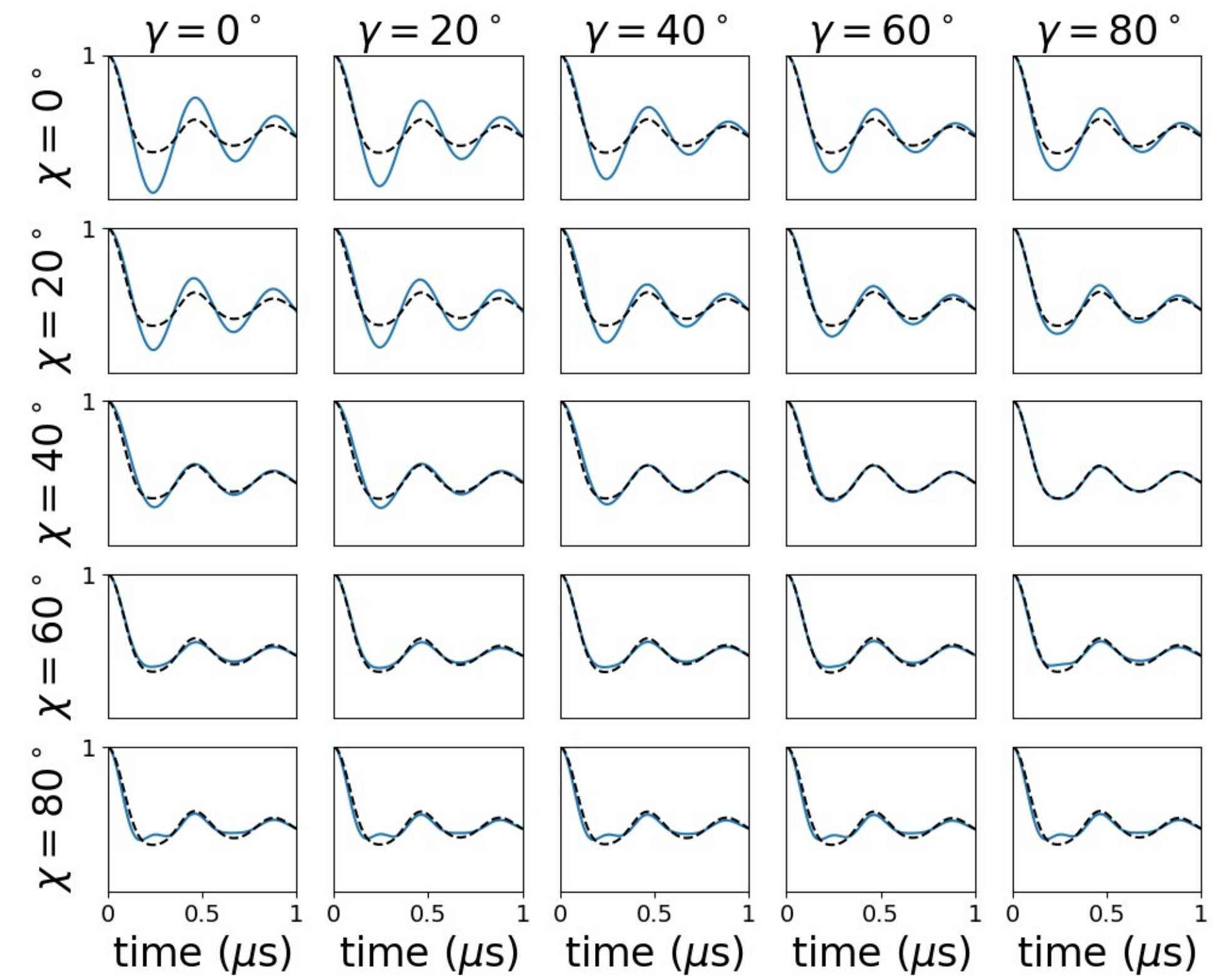

Figure S5: *The simulated and ideal RIDME time traces were obtained for different relative orientations defined with values of χ and γ angles. The value of η is 80°. The standard deviation for each angle was set at 10°. The simulated RIDME was obtained at a field of -125 G from the maximum of the FS-ESE which corresponds to the maximum of the φ-curve as discussed in main text (cf. Figure 3A in the main text for more details).*

φ-Curve Iterations for Each Tested Relative Orientation

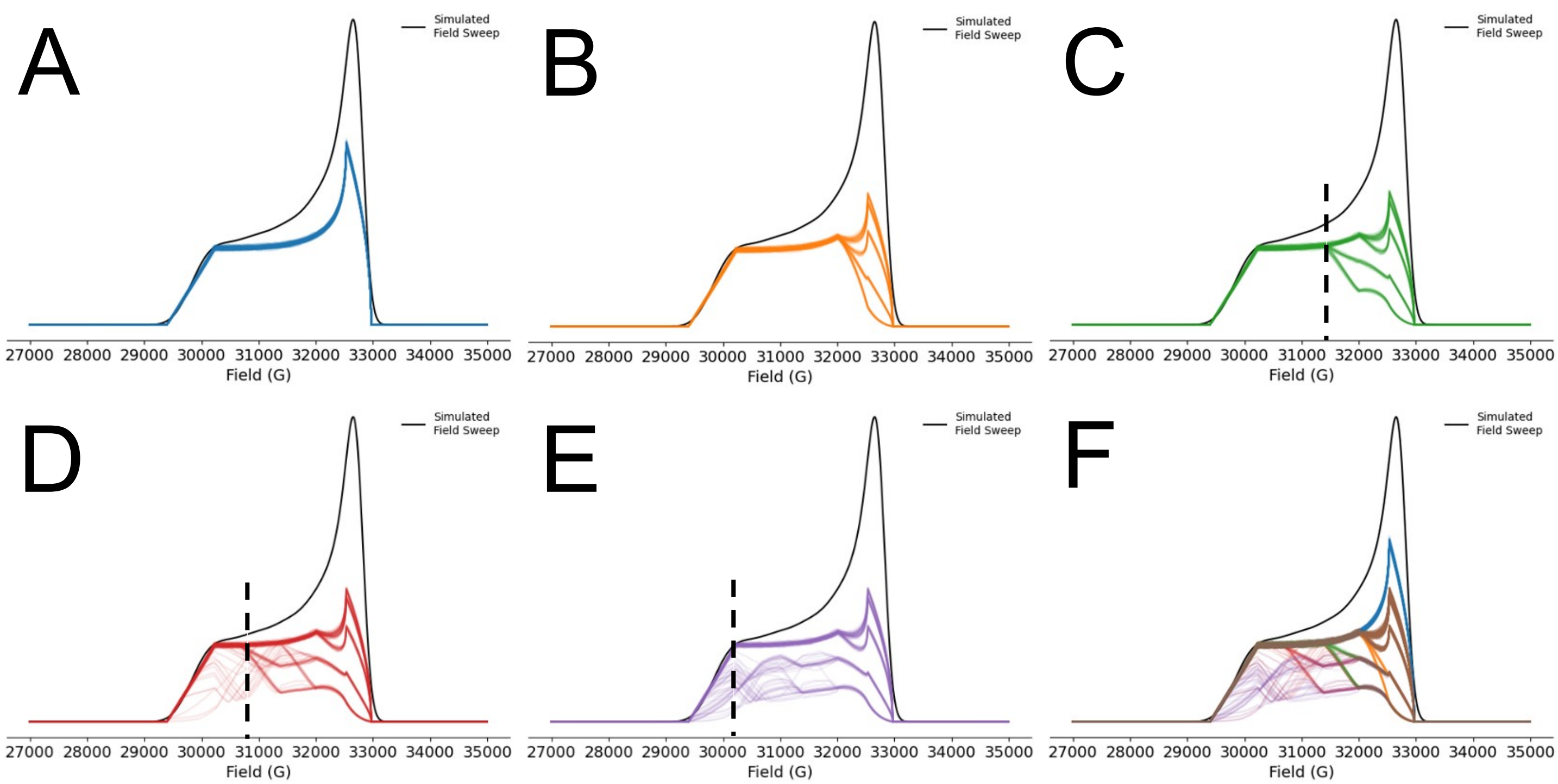

Figure S6: A) *The simulated FS-ESE spectrum (black) overlaid the zeroth φ-curve iteration (blue), calculated for 125 different relative orientations of spin pairs. Each relative orientation gave the same zeroth φ-curve iteration, thus only one line is desirable in blue.* ***B)*** *The first φ-curve iteration (gold), for each of the 125 relative orientations tested.* ***C)*** *The second φ-curve iteration (green) which has a plateau from 30800 G to 31500 G. A dashed black line indicates the position chosen as the third field. The* ***D)*** *third and* ***E)*** *fourth φ-curve iterations similarly plateau, and dashed black lines indicate the field positions chosen for the acquisition scheme.* ***F)*** *The simulated FS-ESE overlaid on all the φ-curve iterations.*

χ^2 Values of Simulated RIDMEs for Each Tested Relative Orientation

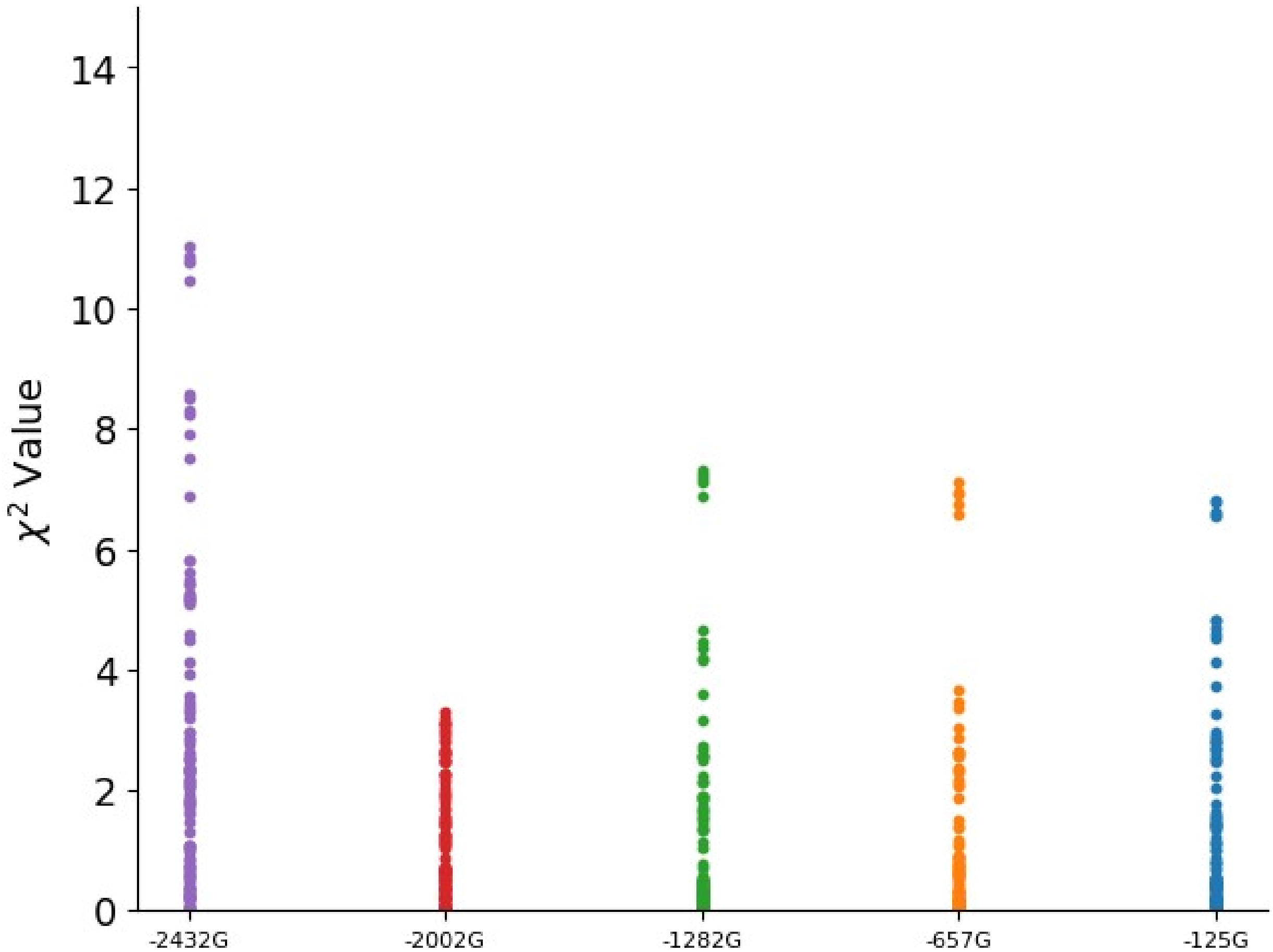

Figure S7: *A scatter plot of χ^2 values between the simulated RIDME and the ideal RIDME signal for each 1-field acquisition scheme of the suggested 5 fields, applied to a systematic sampling of 125 unique relative orientations.*

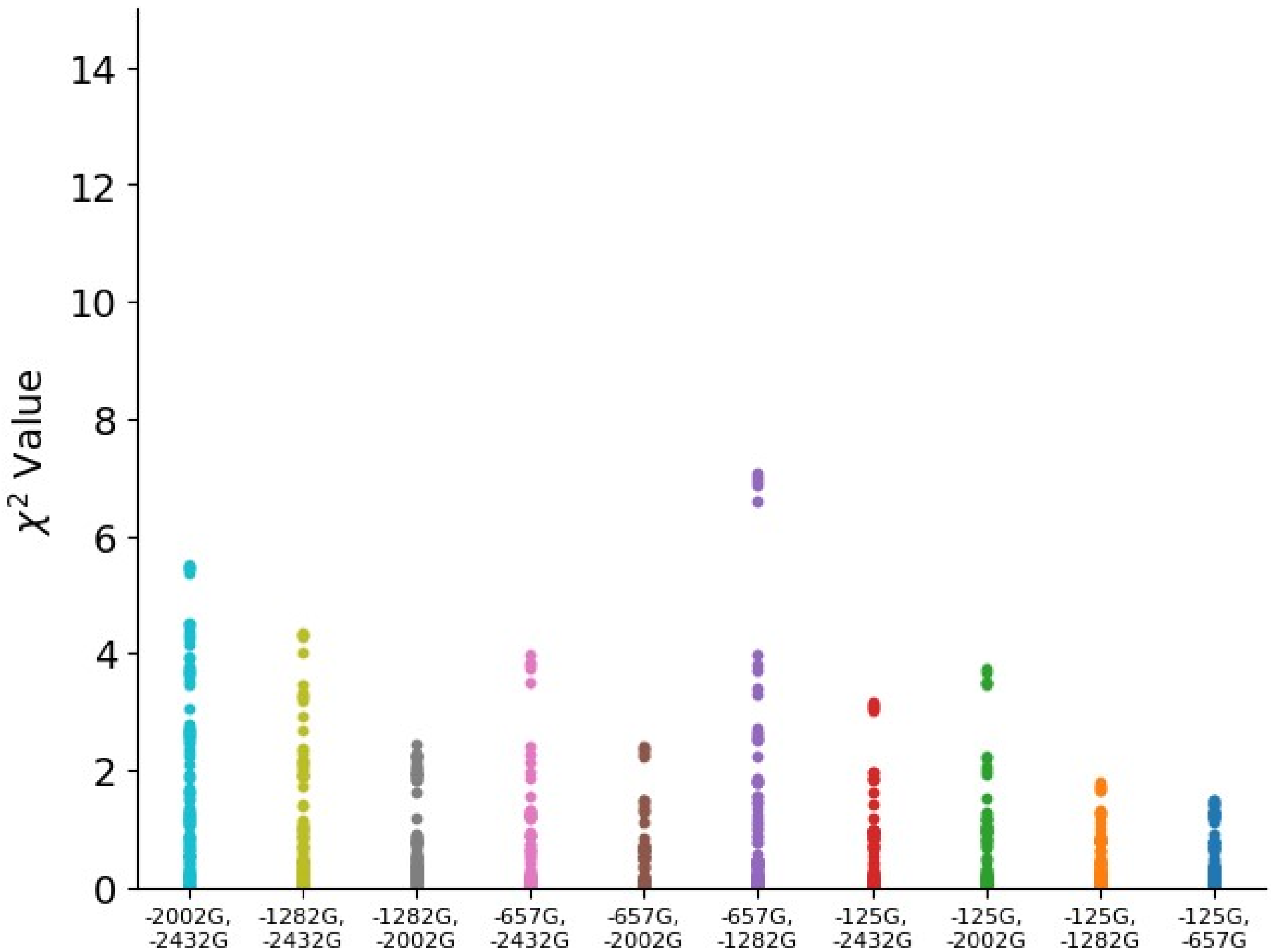

Figure S8: *A scatter plot of χ^2 values between the simulated RIDME and the ideal RIDME signal for each 2-field acquisition scheme of the suggested 5 fields, applied to a systematic sampling of 125 unique relative orientations.*

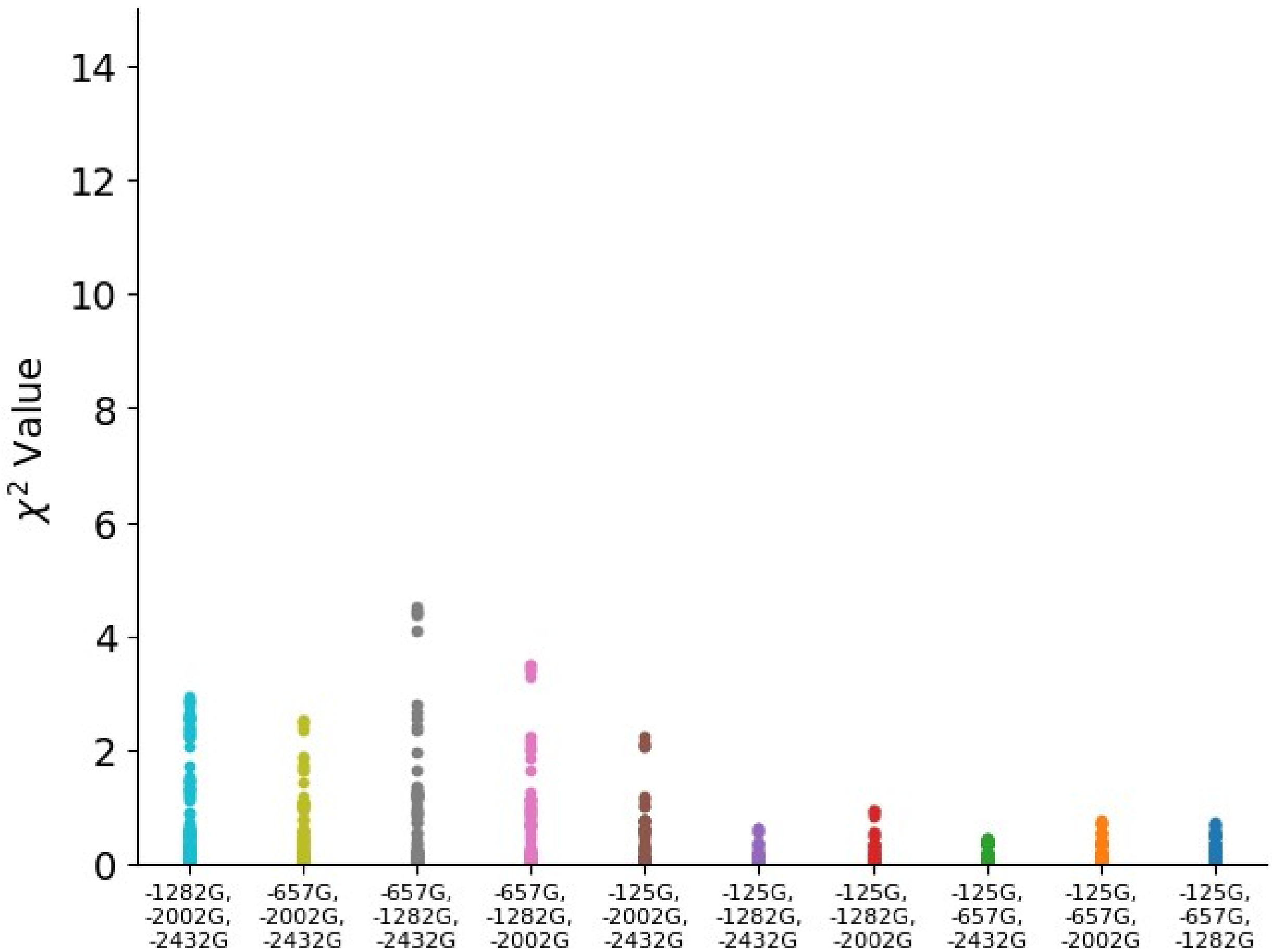

Figure S9: *A scatter plot of χ^2 values between the simulated RIDME and the ideal RIDME signal for each 3-field acquisition scheme of the suggested 5 fields, applied to a systematic sampling of 125 unique relative orientations.*

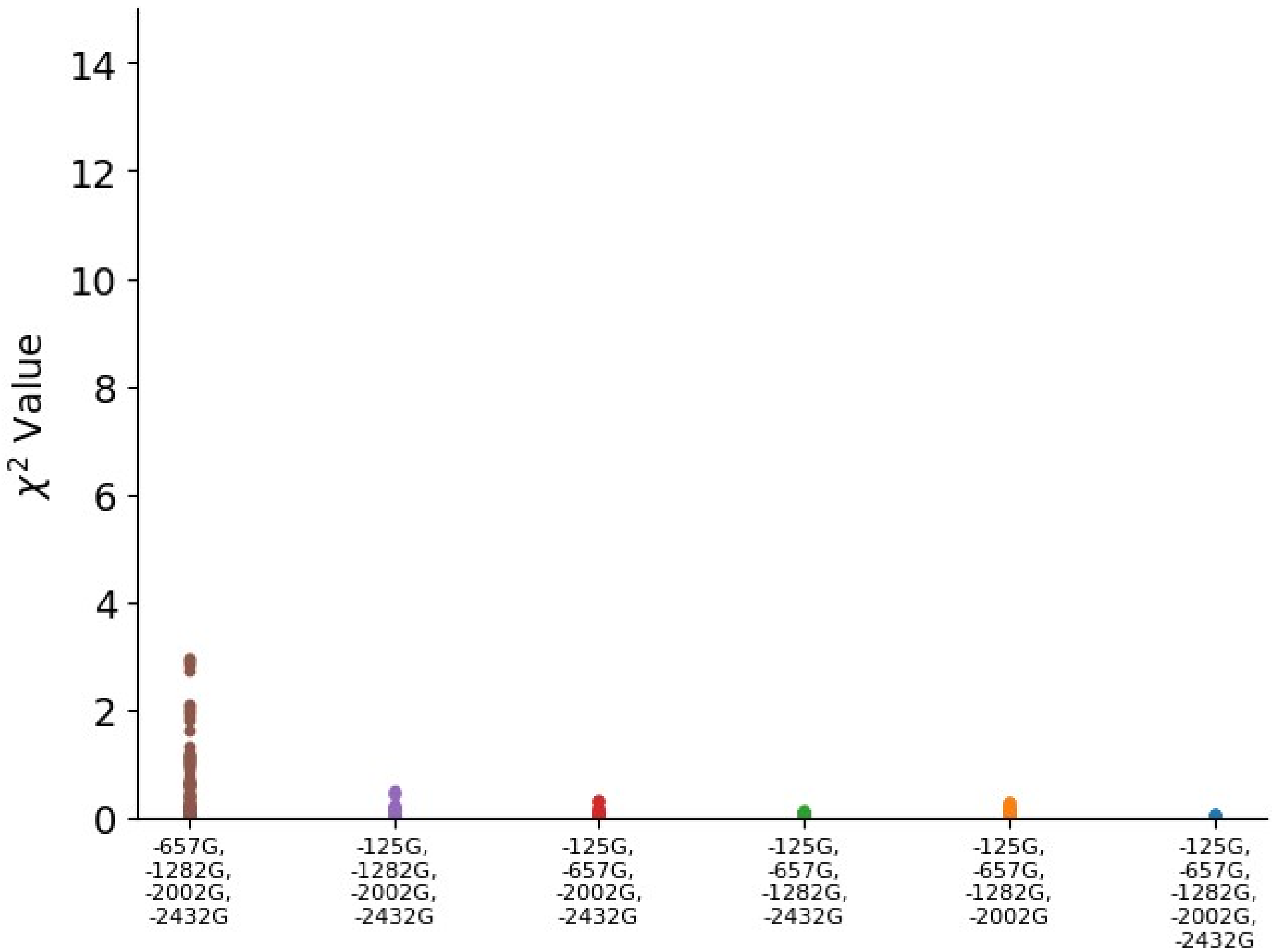

Figure S10: *A scatter plot of χ^2 values between the simulated RIDME and the ideal RIDME signal for each 4-field acquisition scheme of the suggested 5 fields, as well as all five fields (blue, right), applied to a systematic sampling of 125 unique relative orientations.*

Raw Experimental Data with Background Components and Pake Patterns of Background Corrected Data from DEERAnalysis 2022.[1]

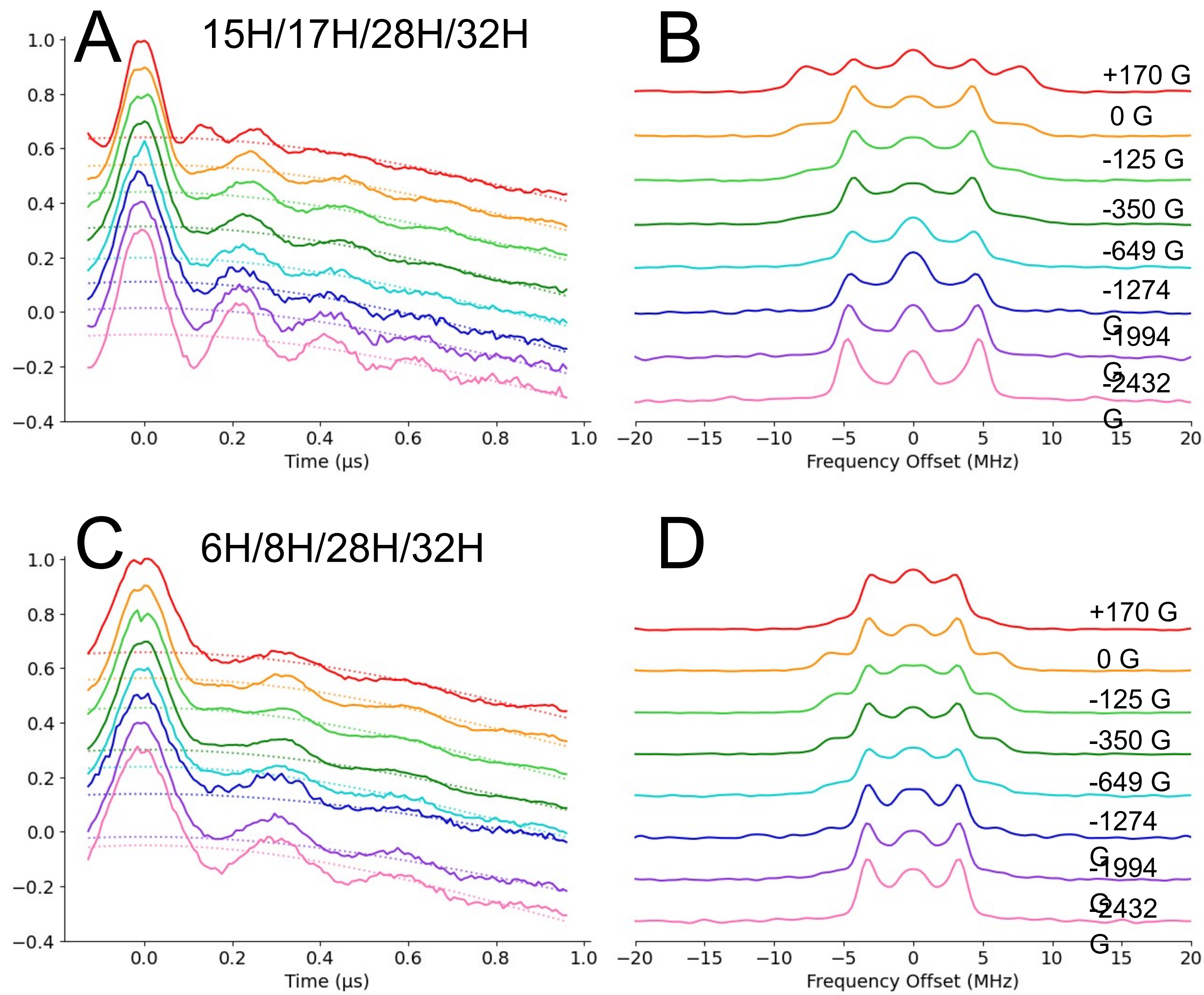

Figure S11 A) *The primary RIDME time traces at each of the eight collected field positions for the 15H/17H/28H/32H GB1 mutant.* ***B)*** *The corresponding Pake patterns for the 15H/17H/28H/32H GB1 mutant.* ***C)*** *The primary RIDME time traces at each of the eight collected field positions for the 6H/8H/28H/32H GB1 mutant.* ***D)*** *The corresponding Pake patterns for the 6H/8H/28H/32H GB1 mutant.*

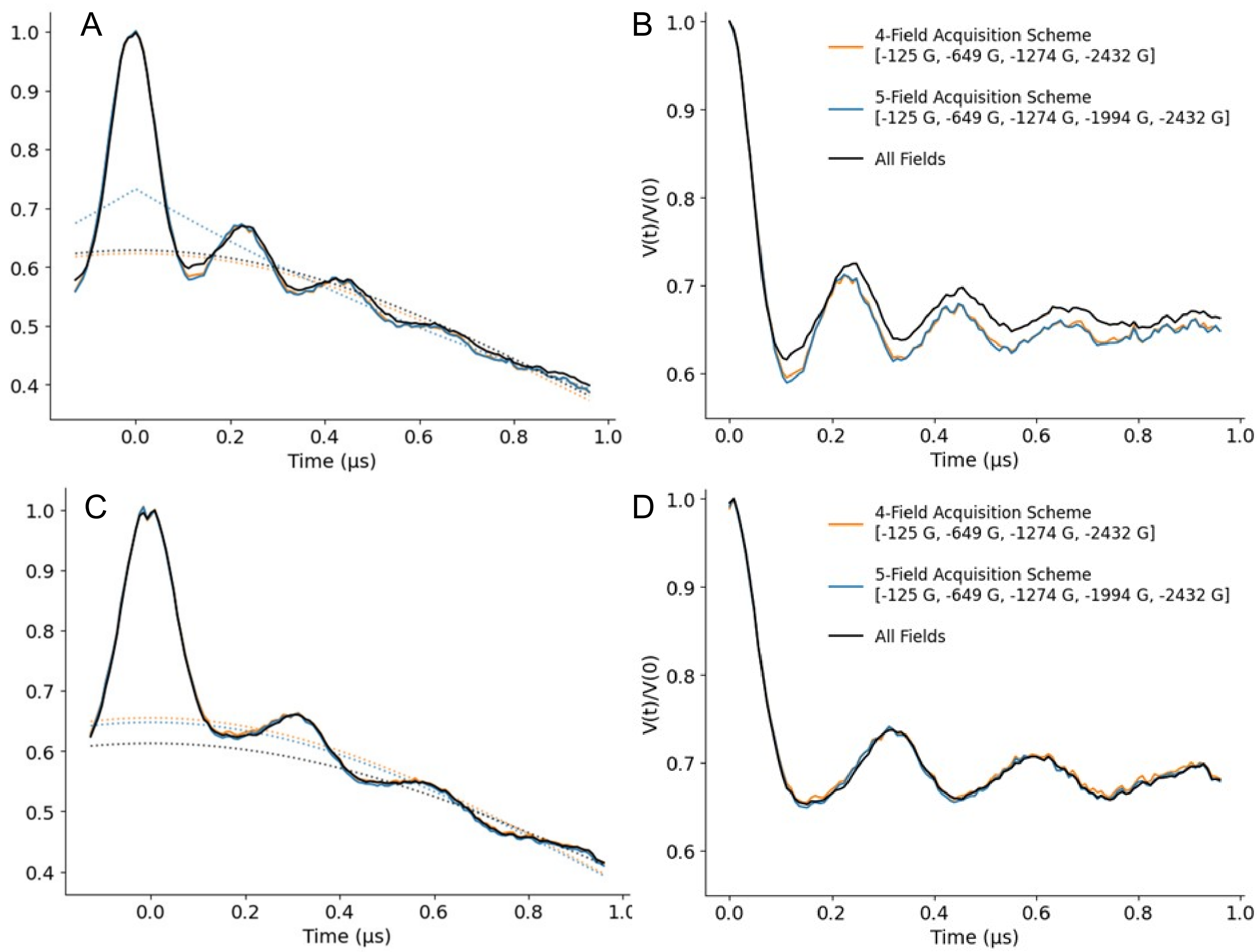

Figure S12: A) *The primary time traces of the 15H/17H/28H/32H GB1 mutant. The sum of all 8 field positions (black), 5 fields (blue), and 4 fields (orange) are shown.* ***B)*** *The corresponding background corrected time traces for the 15H/17H/28H/32H GB1 mutant.* ***C)*** *The raw time traces of the 6H/8H/28H/32H GB1 mutant are shown. The sum of all 8 field positions (black), 5 fields (blue), and 4 fields (orange are displayed).* ***D)*** *The corresponding background corrected time traces for the 6H/8H/28H/32H GB1 mutant.*

DEERNet[2] Time Trace Analysis

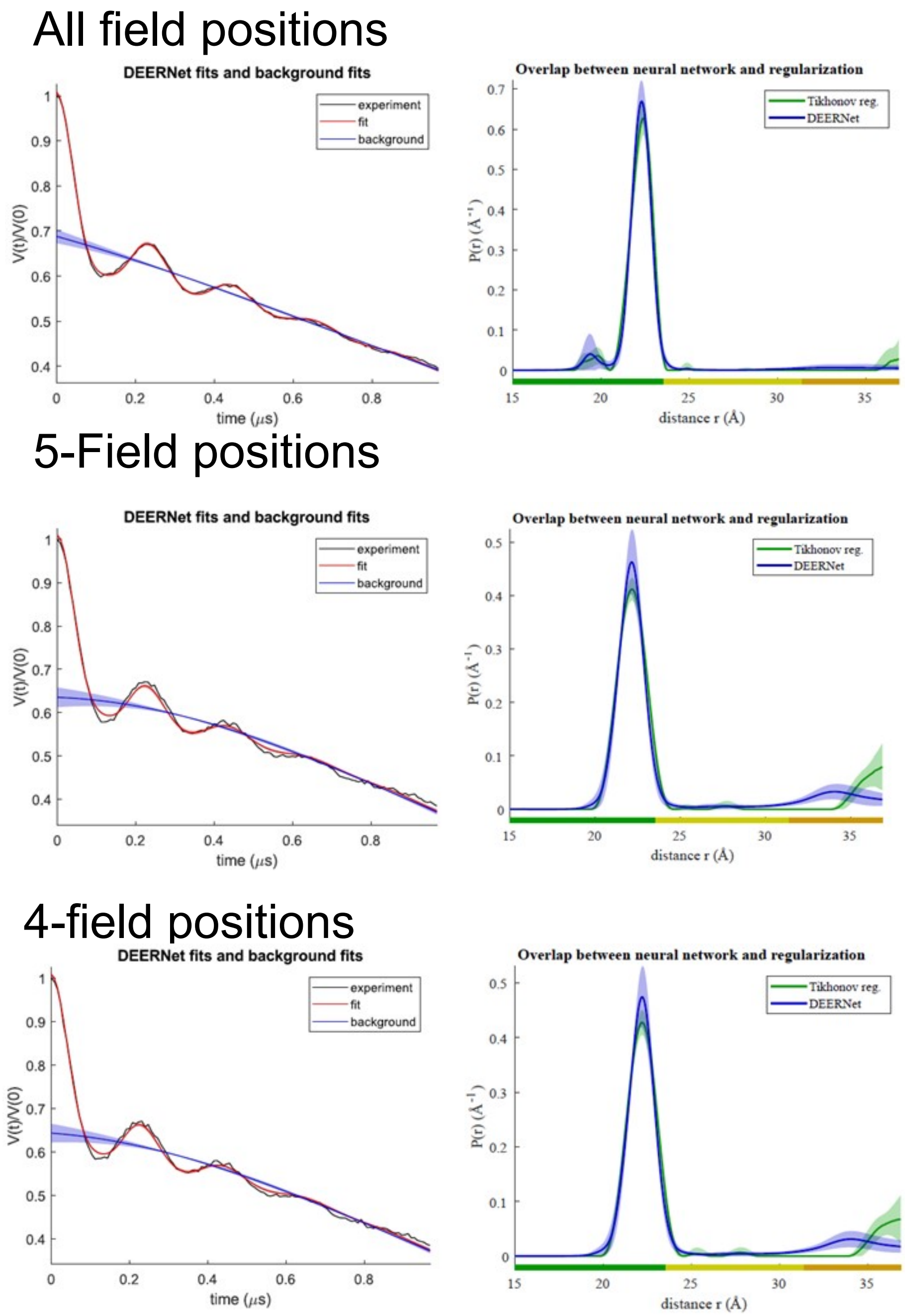

Figure S13: *The DEERNet analysis of the various acquisition schemes for the 15H/17H/28H/32H GB1 mutant. The data is for the sum of all 8 field positions (top left), with the resulting distance distribution overlaid with the distance distribution found via Tikhonov regularization (top right). The DEERNet analysis of the 5-field acquisition scheme (middle left), with the corresponding distance distribution, overlaid with the distance distribution found via Tikhonov regularization (middle right). The DEERNet analysis of the 4-field acquisition scheme (bottom left), with the corresponding distance distribution, overlaid with the distance distribution found via Tikhonov regularization (bottom right).*

All field positions

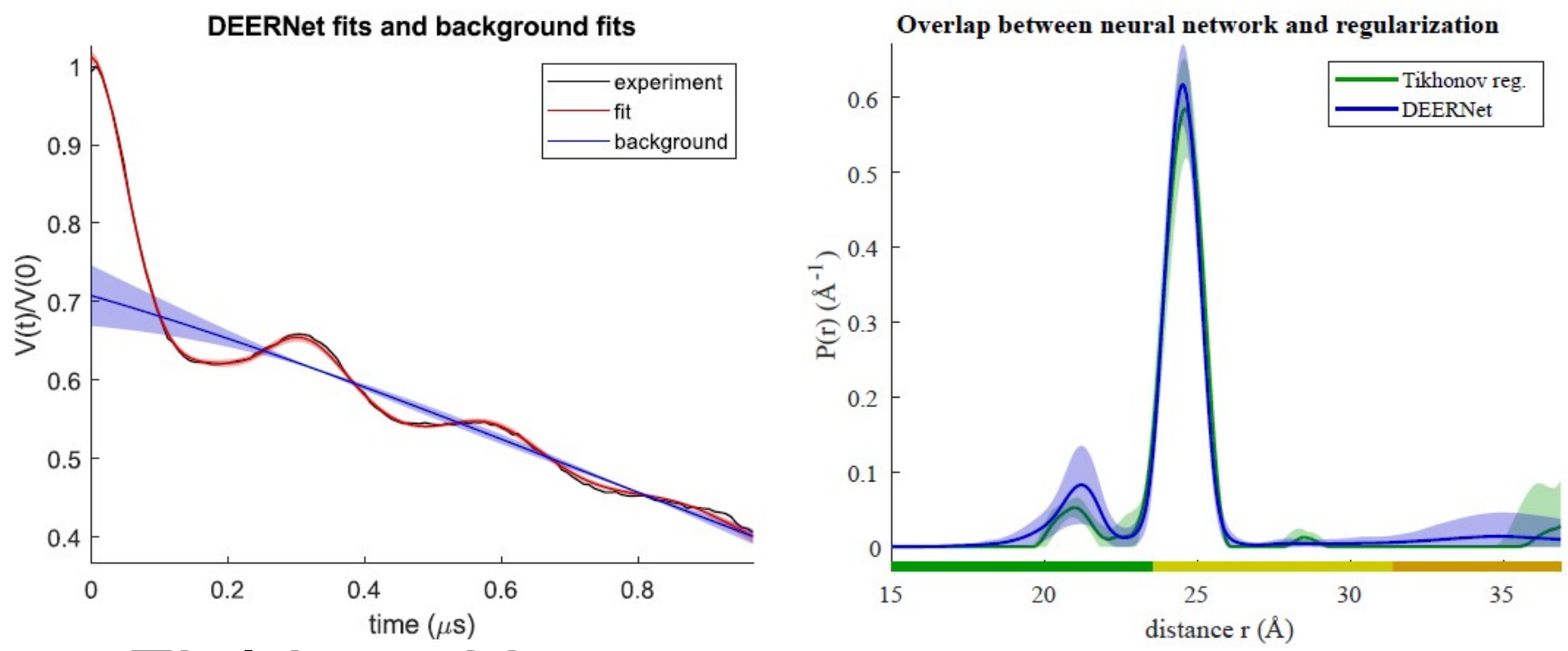

5-Field positions

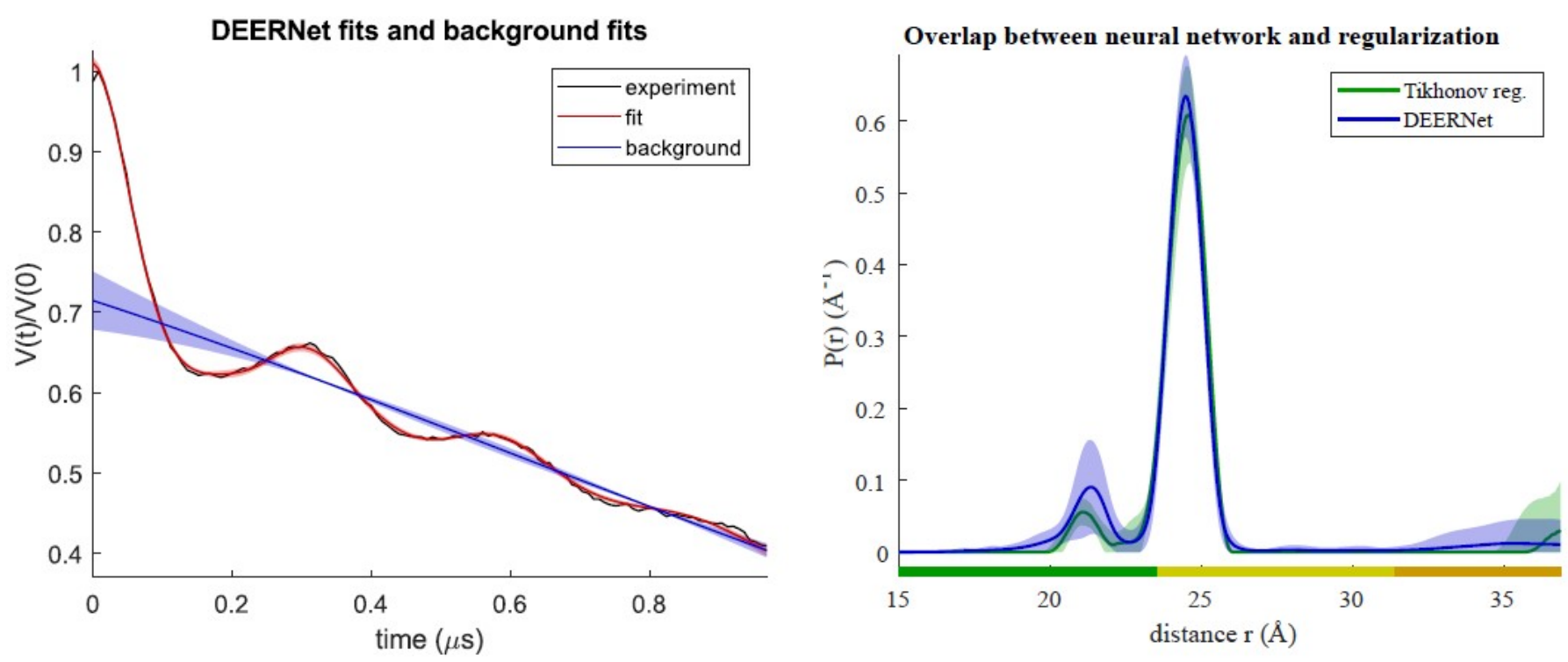

4-field positions

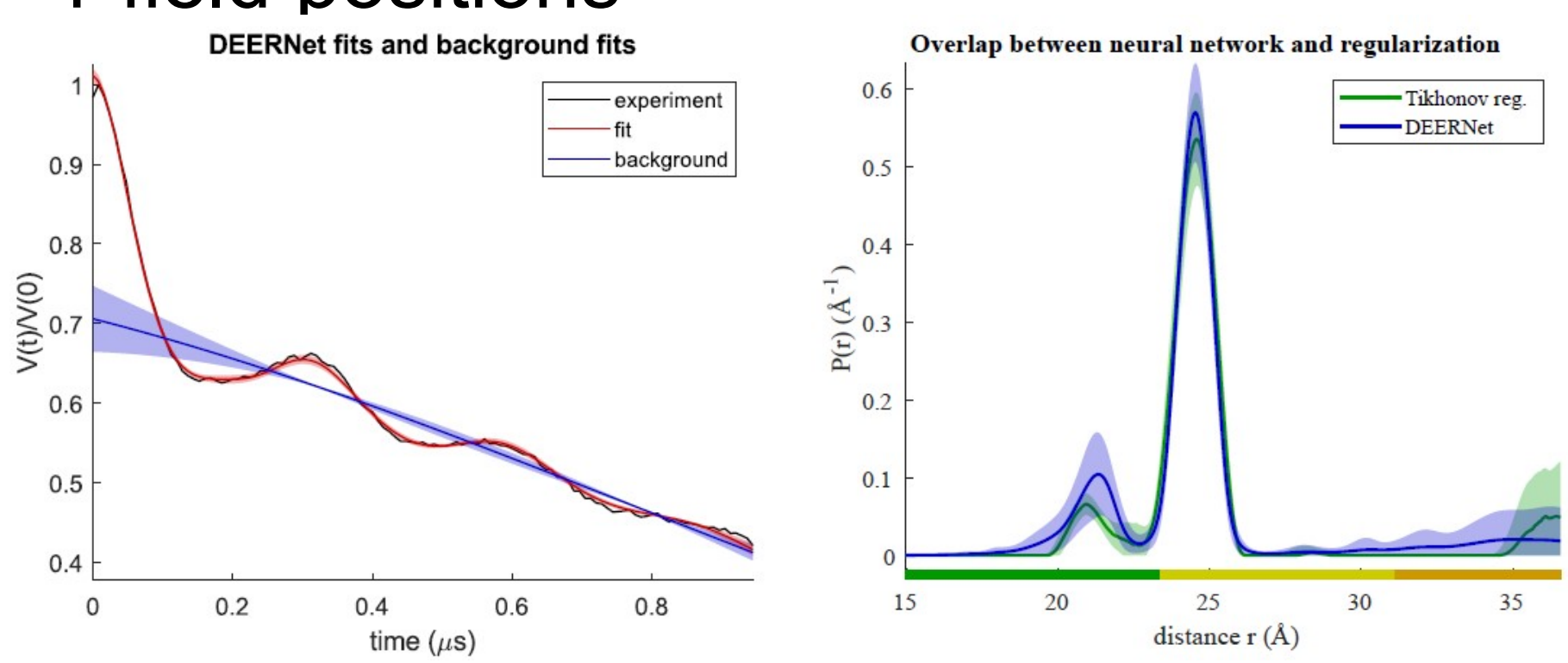

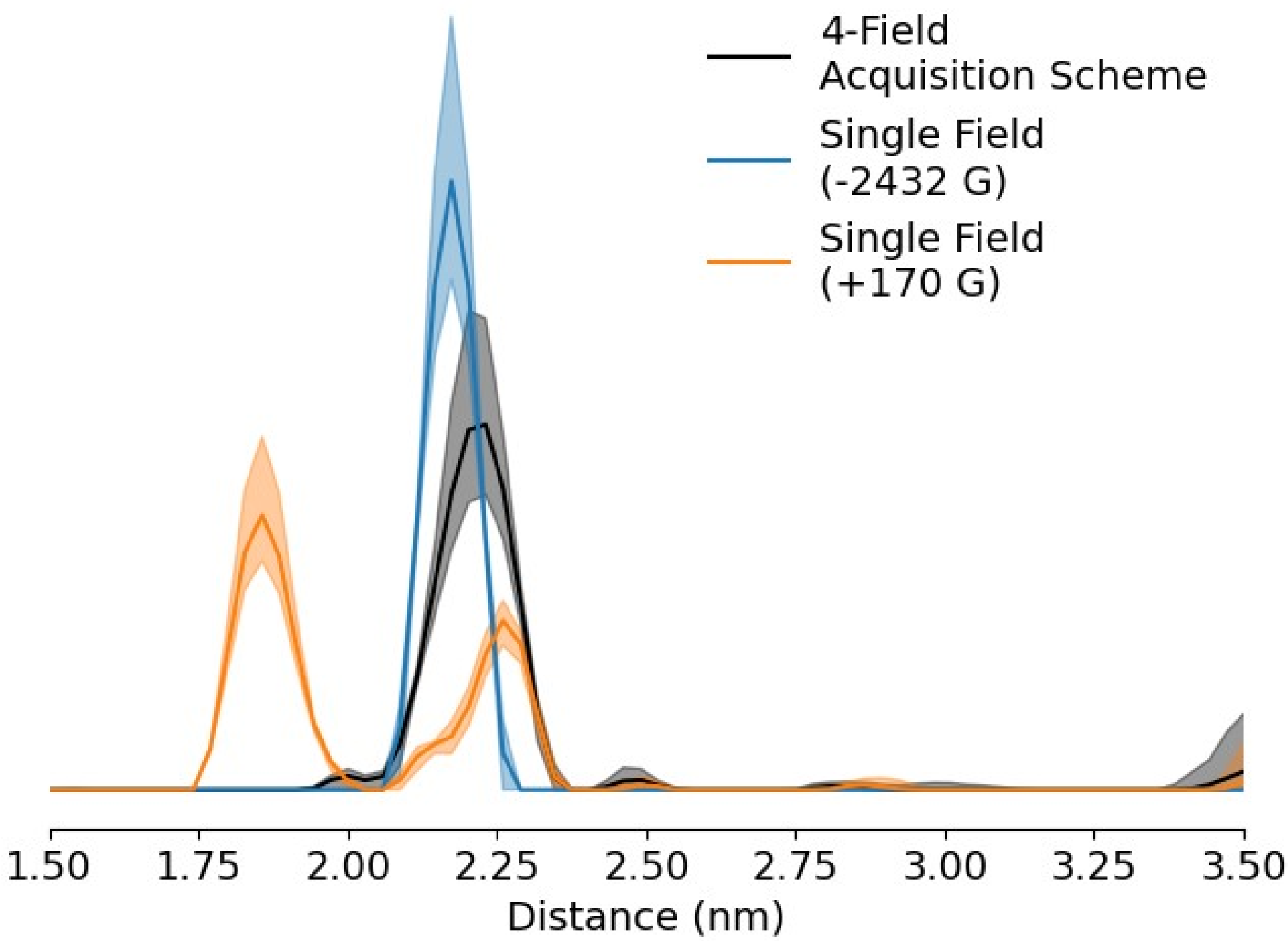

Figure S15: *The distance distributions from the weighted average time domain signals for the optimal 4-field (black) and from single field acquisitions at -2432 G (blue) and +170 G (orange) collected on the E15H/T17H/K28H/Q32H GB1 mutant. The shaded area is the 95% confidence interval calculated by varying the background subtracted component. Notably the mean distance is shifted down by 0.5 Å and narrowed for the -2432 G field data due to oversampling of θ = 90°. A bimodal distribution is observed with the +170 G field data due to oversampling of θ = 0°.*

Refrences

1. L. Fábregas-Ibáñez, M. H. Tessmer, G. Jeschke and S. Stoll, Phys Chem Chem Phys, 2022, 24, 2504–2520.
2. S. G. Worswick, J. A. Spencer, G. Jeschke and I. Kuprov, Sci Adv, 2018, 4, eaat5218.
